# PLA2G4A promotes right-sided colorectal cancer progression by inducing CD39+γδ Treg polarization

**DOI:** 10.1172/jci.insight.148028

**Published:** 2021-08-23

**Authors:** Yang Zhan, Lei Zheng, Jia Liu, Dongzhi Hu, Junfeng Wang, Kai Liu, Jiansheng Guo, Ti Zhang, Dalu Kong

**Affiliations:** 1 Tianjin Medical University Cancer Institute and Hospital, National Clinical Research Center for Cancer, Tianjin’s Clinical Research Center for Cancer, Key Laboratory of Cancer Prevention and Therapy, Tianjin, China.; 2 Department of Hepatic Surgery, Fudan University Shanghai Cancer Center, Shanghai Medical College, Fudan University, Shanghai,China.

**Keywords:** Oncology, Colorectal cancer

## Abstract

The **γδ** T cell is a promising candidate cell in tumor immunotherapy. However, **γδ** T cells polarize to CD39^+^**γδ** Tregs upon colorectal cancer (CRC) induction, and the underlying mechanism remains unclear. Here, we show that the frequency of CD39^+^**γδ** Tregs, which positively correlated with poor prognosis, was significantly higher in right-sided CRC (RSCRC) than in the left-sided CRC (LSCRC). Interestingly, CD39^+^**γδ** Tregs from RSCRC showed stronger immunosuppressive phenotype and function than LSCRC. Furthermore, the quantitative mass spectrometry data show that CD39^+^**γδ** Treg polarization was related to the abnormal activation of the Phospholipase a2-IVa/Arachidonic acid (PLA2G4A/AA) metabolic pathway in RSCRC. Using an in vitro coculture system and an orthotopic murine model of CRC, we show that the overexpression of *Pla2g4a* in CT26 cells induced CD39^+^**γδ** Tregs, inhibiting the antitumor immune response. Finally, we found that the overall survival of the PLA2G4A^hi^ group was significantly shortened compared with PLA2G4A^lo^ RSCRC, while the survival of LSCRC showed the opposite. Collectively, RSCRC with abnormal PLA2G4A expression educates **γδ** T cells into CD39^+^**γδ** Tregs to promote tumor progression and metastasis. Our work highlights the interaction between cancer cells and immune cells by distinguishing the primary tumor site and deepens the understanding of the tumor microenvironment and immunosuppression.

## Graphical abstract

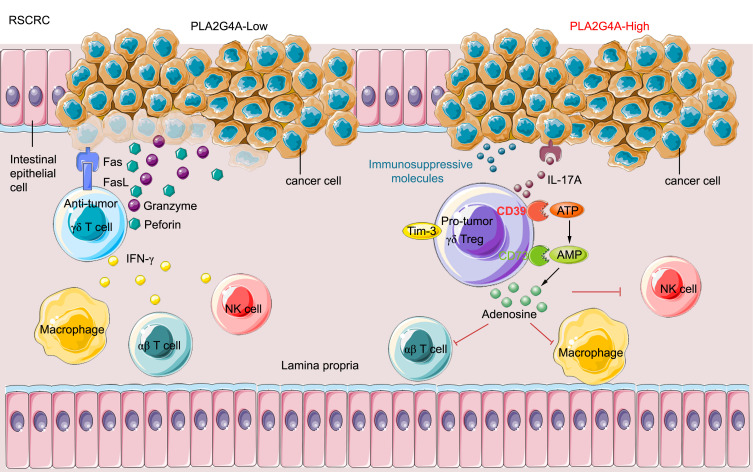

## Introduction

Colorectal cancer (CRC) ranks the third among the most common cancers and has become the second leading cause of cancer death in the world (1). The 5-year relative survival rate was 11.7% for patients with distant metastasis (2). Although immunotherapy, especially PD-1/PD-L1 blockades, has achieved great success in advanced melanoma, non–small cell lung cancer, and Hodgkin’s lymphoma, it was only effective in 4% of patients with metastasis CRC (mCRC) (3). Tumor cells establish an immunosuppressive microenvironment to evade immune surveillance, which becomes the major obstacle in immunotherapy, especially in solid tumors, such as CRC (4). Therefore, a better understanding of the immunosuppressive microenvironment in CRC is critical.

Prognostic stratification based on molecular classification is expected to make treatment for patients more individualized. Many studies have confirmed that there are significant differences in embryonic origin, anatomical supply, clinical manifestations, and molecular genetics of the left-sided CRC (LSCRC) and right-sided CRC (RSCRC) (5–7). The left hemicolon originates from the posterior intestine of the embryo and is supplied by the submesenteric artery. The right hemicolon originates from the central intestine and is supplied by the superior mesenteric artery. LSCRC is common in young male patients, while RSCRC is common in old female patients. The primary tumor site is also an independent predictor of anti-EGFR and anti-VEGF treatment, and a predictor of prognosis of stage IV CRC (8–10). Venook’s CALGB/SWOG 80405 clinical data were presented in ASCO in 2016 and showed that the survival of patients with RSCRC is significantly shortened compared with stage III–IV LSCRC (11). The latest research confirmed that the primary tumor location significantly impacts CRC prognosis (12). In 2017, the national comprehensive cancer network (NCCN) guidelines recommended that the primary tumor site should be included in the reference basis for the selection of targeted drugs in the first-line treatment of advanced CRC (13). In 2019, NCCN issued guidelines for diagnosing and treating CRC, which indicated the differences between LSCRC and RSCRC medication (cetuximab is no longer recommended for preoperative conversion therapy for advanced RSCRC). However, the current research has not explored the molecular mechanism of the difference between the medications and could not break through the existing treatment. Therefore, it is urgent to explore the molecular mechanism of the differences in LSCRC/RSCRC progression.

Tregs are vital immunosuppressive cells that contribute to suppressing immune responses, mediating immune tolerance, and facilitating tumorigenesis and metastasis. Although traditional αβ TCR–presenting Tregs, such as CD4^+^ or CD8^+^ Tregs, have been extensively studied (9), much less is known about intratumor γδ Tregs. Recently, a potentially novel CD39^+^γδ Treg in human CRC has been identified (14). CD39^+^γδ Tregs are the predominant Tregs in CRC and mediate direct and robust immunosuppressive effects on CD3^+^ T cells via the adenosine pathway — but independently of TGF-β or IL-10 (14). However, the roles and underlying mechanism of CD39^+^γδ Tregs in the immunosuppressive microenvironment of LSCRC/ RSCRC remain unclear.

In this study, we reveal that a higher accumulation of tumor-infiltrating CD39^+^γδ Tregs in RSCRC compared with paired normal tissue and LSCRC, along with increased production of IL-17A and adenosine, enhanced the immunosuppressive function. Furthermore, phospholipase a2-IVa/Arachidonic acid (PLA2G4A/AA) metabolic pathway was abnormally activated in RSCRC, which induced CD39^+^γδ Treg polarization and indicated a poor prognosis.

## Results

### The frequency of tumor-infiltrating CD39^+^γδ Tregs was significantly increased in RSCRC compared with LSCRC. 

**Figure 1:**
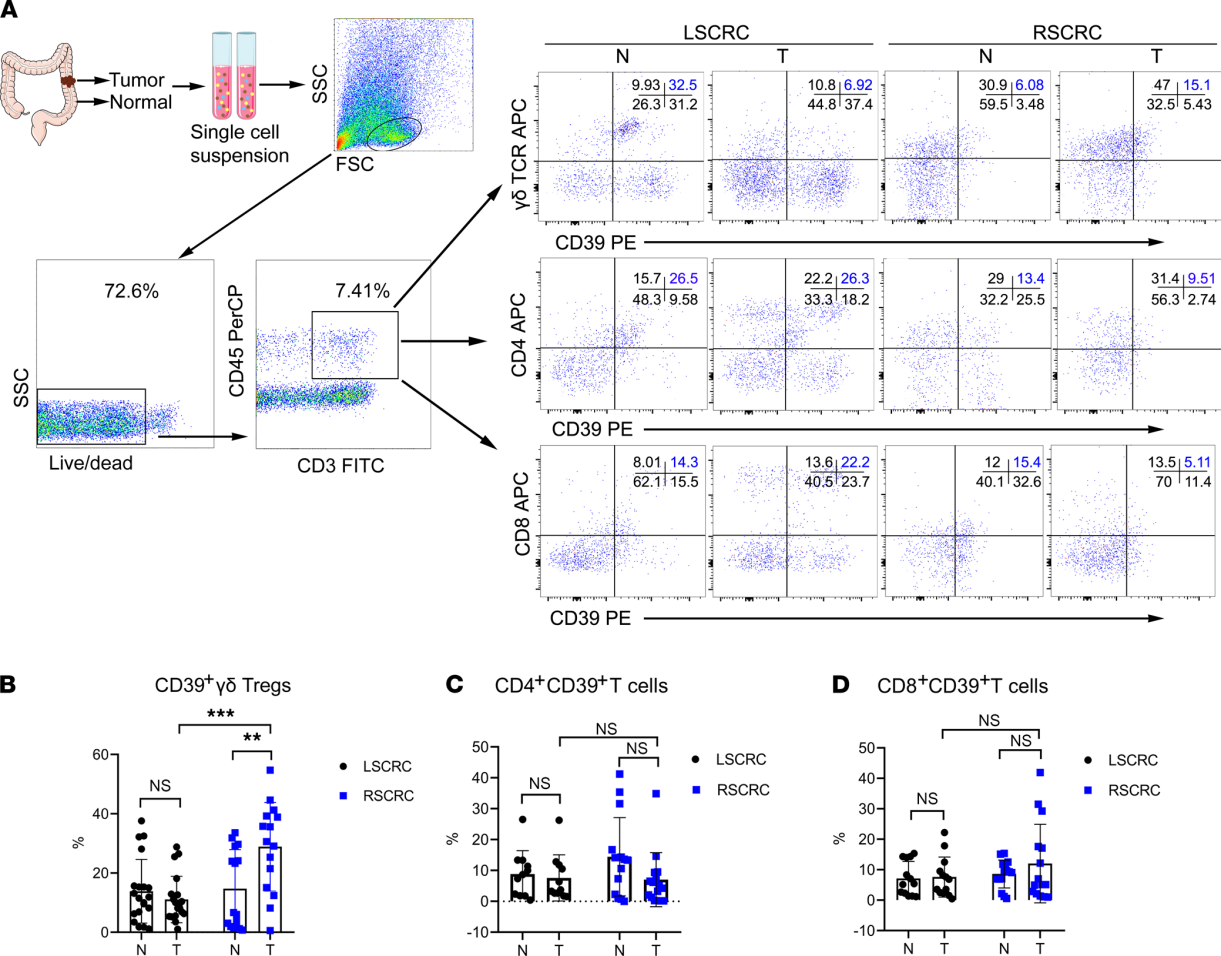
The infiltration of CD39+γδ Tregs was significantly increased in RSCRC compared with LSCRC. (**A**) Representative flow cytometric analysis of the distribution of CD39^+^ Tregs in CRC and paired normal tissues. (**B**–**D**) The percentages of CD39^+^ γδ Tregs (**B**), CD4^+^ T cells (**C**), and CD8^+^ T cells (**D**) in the total tumor-infiltrating CD3^+^ T cells was calculated. Data are shown as mean ± SEM, using 2-way ANOVA followed by Sidak’s multiple-comparisons test. RSCRC, *n* = 15; LSCRC, *n* = 20. Each dot represents 1 patient. N, normal; T, tumor; RSCRC, right-sided colorectal cancer; LSCRC, left-sided colorectal cancer. **P < 0.01, ***P < 0.001.****

The CD39 adenosine pathway is important in regulating the immune system and tumor immunosuppressive microenvironment (15–17). A previous study has identified CD39^+^γδ T cells as a subset of Tregs with strong immunosuppressive functions, and they correlated positively with malignant clinicopathological features in human CRC (14). However, the distribution and functional changes in different tumor microenvironments remain unclear. In the present study, we found that the frequencies of CD39^+^γδ Tregs were changed between RSCRC and LSCRC. Compared with the paired normal control (14.72% ± 13.21%), the frequency of tumor-infiltrating CD39^+^γδ Tregs was significantly increased in RSCRC (28.87% ± 14.94%), while it was decreased in LSCRC (11.08% ± 7.83%, *P* < 0.001, *n* = 35; Figure 1, A and B). In addition, the infiltration of CD4^+^CD39^+^ T cells tended to decrease (normal versus tumor = 14.43% ± 12.67% versus 7.01% ± 8.73%; *P* = 0.22; Figure 1, A and C) and the frequency of CD8^+^CD39^+^ T cells (normal versus tumor = 8.60% ± 4.58% versus 12.02% ± 12.92%, *P* = 0.67; Figure 1, A and D) tended to increase in RSCRC. Notably, these tumor-infiltrating γδ T cells (γδ-TILs) were dominantly of the Vδ1 subset, indicating that they are derived from intestinal tissue–resident γδ T cells (Supplemental Figure 1; supplemental material available online with this article; https://doi.org/10.1172/jci.insight.148028DS1).

### Differentiation phenotype of CD39^+^γδ Tregs in RSCRC/LSCRC.

**Figure 2:**
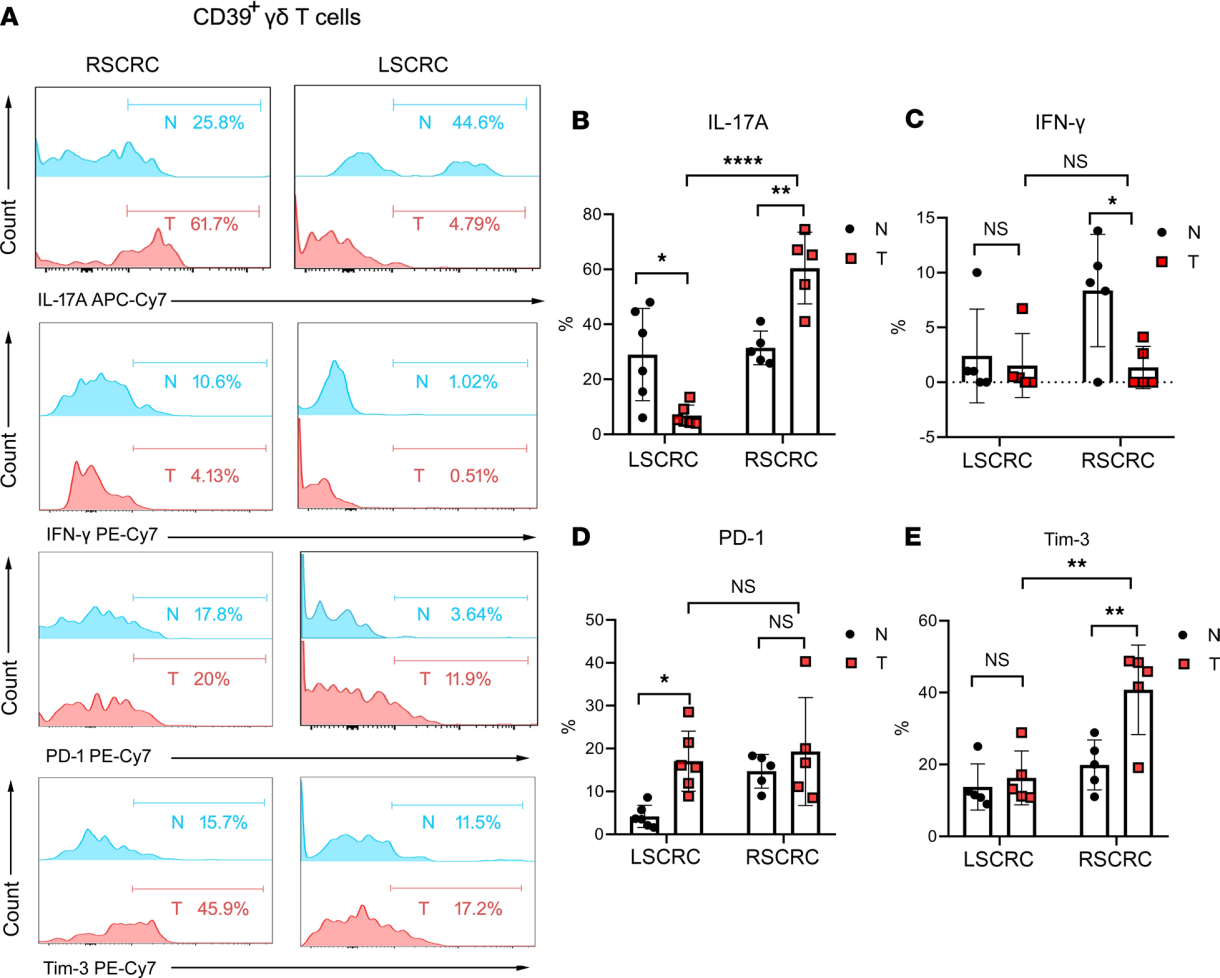
Differentiation phenotype of CD39+γδ Tregs in RSCRC/LSCRC. (**A**) Representative histogram plot of the expression of IL-17A/IFN-γ/PD-1/Tim-3 in/on CD39^+^ Tregs derived from LSCRC/RSCRC. (**B**–**E**) The percentages of IL-17A (**B**), IFN-γ (**C**), PD-1 (**D**), and Tim-3 (**E**) in CD39^+^γδ T cells was calculated. Data are shown as mean ± SEM, using 2-way ANOVA followed by Sidak’s multiple-comparisons test. RSCRC, *n* = 5; LSCRC, *n* = 6. Each dot represents 1 patient. N, normal; T, tumor; RSCRC, right-sided colorectal cancer; LSCRC, left-sided colorectal cancer. *P < 0.05, **P < 0.01, ****P < 0.0001.

The antitumor function of γδ T cells is mainly based on IFN-γ production (18), while the protumor function is linked to IL-17 production (19–21). Thus, we measured the productions of these 2 cytokines in tumor-infiltrating CD39^+^γδ Tregs. The flow cytometry results showed that IL-17A–producing CD39^+^γδ Tregs in RSCRC were dramatically increased compared with the paired normal tissues (60.46% ± 5.82% versus 31.44% ± 2.74%, respectively; *P* = 0.004), while significant downregulation of IL-17A was detected in CD39^+^γδ Tregs in LSCRC (6.85% ± 1.54%; Figure 2, A and B). Compared with normal tissues, the level of IFN-γ–producing CD39^+^γδ Tregs in RSCRC was decreased (8.36% ± 2.29% versus 1.35% ± 0.86%, respectively; *P* = 0.043) and showed no change in LSCRC (Figure 2, A and C). These data indicate the stronger protumor function of CD39^+^γδ Tregs in RSCRC than their left-sided counterpart, and that may relate to the worse prognosis of patients with RSCRC.

Next, we detected the expression of immune checkpoint molecules (22, 23) on CD39^+^γδ Tregs in different primary tumor sites by flow cytometry. Interestingly, the expression of PD-1 was significantly increased on CD39^+^γδ Tregs in LSCRC compared with that in paired normal tissues (17.06% ± 2.87% versus 4.19% ± 1.06%, respectively; *P* = 0.033), while a substantial increase of T cell immunoglobulin mucin family member 3 (Tim-3) was found on CD39^+^γδ Tregs in RSCRC (40.76% ± 12.44%; Figure 2, D and E). These data show the different phenotypes of CD39^+^γδ Tregs in RSCRC/LSCRC. Thus, the target of cancer immunotherapy for patients with RSCRC/LSCRC should be distinguished.

### CD39^+^γδ Tregs in RSCRC showed enhanced immunoregulatory function and positively correlated with malignant clinicopathological features.

**Figure 3:**
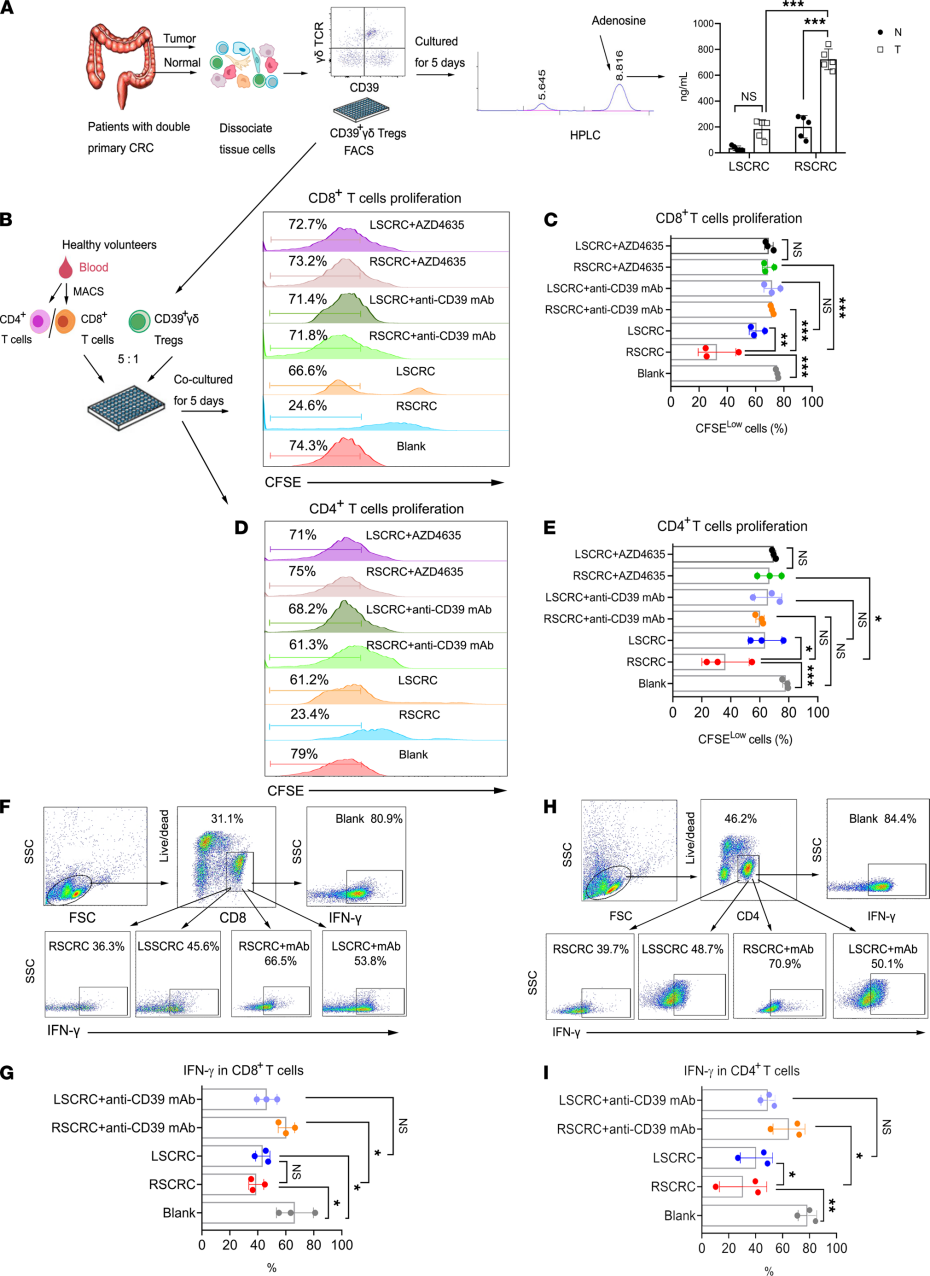
CD39+γδ Tregs derived from RSCRC showed enhanced immunoregulatory function compared with LSCRC. (**A**) HPLC assay to measure adenosine concentrations in the supernatants of CD39^+^γδ Tregs on day 5. Data are shown as mean ± SEM, using 2-way ANOVA followed by Sidak’s multiple-comparisons test; *n* = 5/group. Sorted CD39^+^γδ Tregs from RSCRC and LSCRC were in vitro cocultured with CFSE-labeled allogeneic CD4^+^/CD8^+^ T cells in the presence of CD3 and CD28 mAbs. (**B** and **D**) Representative histogram plot of CD8^+^ T cells and CD4^+^ T cells. (**C** and **E**) Bar diagram summarizes the percentages of proliferated cells (CFSE^lo^) in CD8^+^ T cells (**C**) and CD4^+^ T cells (**E**). (**F** and **H**) Representative flow cytometric analysis of IFN-γ in CD8^+^ T cells (**F**) and CD4^+^ T cells (**H**). (**G** and **I**) The percentages of IFN-γ^+^ cells in CD8^+^ T cells (**G**) and CD4^+^ T cells (**I**) was summarized. One-way ANOVA followed by Tukey’s multiple-comparison test was used for multiple-group analysis. Data are shown as mean ± SEM. RSCRC, *n* = 3; LSCRC, *n* = 3 from 3–5 independent experiments. *P < 0.05; **P < 0.01; ***P < 0.001.

It has been proven that the inhibitory effect of CD39^+^γδ Tregs on effector T cells is achieved by direct secretion of adenosine (14). Therefore, we next sorted tumor-infiltrating CD39^+^γδ Tregs from patients with double primary CRC (RSCRC and LSCRC) and then detected adenosine in the supernatant by high-performance liquid chromatography (HPLC) after being cultured for 5 days. We found that the adenosine level secreted by CD39^+^γδ Tregs in RSCRC was significantly higher than that in the left counterpart (*P* < 0.01, *n* = 5). Compared with paired normal tissues, CD39^+^γδ Tregs in RSCRC secreted much more adenosine in the supernatant (Figure 3A). In order to determine the direct suppression of CD39^+^γδ Tregs on αβ T cell proliferation, CD39^+^γδ Tregs were sorted from patients with RSCRC/LSCRC and then were in vitro cocultured with CD4^+^/CD8^+^ T cells isolated from PBMC of healthy volunteers in the presence of CD3 and CD28 mAbs. As shown in Figure 3, B–E, tumor-infiltrating CD39^+^γδ Tregs in RSCRC significantly inhibited both CD4^+^/CD8^+^ T cell proliferation, and as expected, their suppressive functions were stronger than those of LSCRC. Using the neutralizing antibody to suppress CD39 or blocking adenosine receptor signals both rescued the proliferation of αβ T cells. Also, CD39^+^γδ Tregs in LSCRC and RSCRC both reduced the level of IFN-γ in CD8^+^ T cells, and inhibiting CD39 rescued IFN-γ production (Figure 3, F and G). Compared with LSCRC, the CD39^+^γδ Treg in RSCRC significantly suppressed the production of IFN-γ by CD4^+^ T cells (Figure 3, H and I). Tregs need the continuous stimulation of various immunosuppressive cytokines and small molecule metabolites to maintain their functions. Thus, we further explored whether adenosine levels altered among different sites led to the functional differences of CD39^+^γδ Tregs from LSCRC/RSCRC. As shown in Supplemental Figure 2, adenosine level was dramatically increased in ascending colon cancer (790.2 ± 178.9 μΜ/L) compared with descending colon cancer (250.9 ± 78.7 μΜ/L), sigmoid colon (173.0 ± 40.1 μΜ/L), and rectal cancer (355.7 ± 83.8 μΜ/L).

Given the substantial immunoregulatory function of CD39^+^γδ Tregs in RSCRC, we next analyzed the correlation between CD39^+^γδ Treg expression and clinicopathological parameters of CRC. As shown in Table 1, the frequencies of tumor-infiltrating CD39^+^γδ Tregs were positively associated with lymph node metastasis and tumor stage (*P* = 0.01). These findings suggest that tumor-infiltrating CD39^+^γδ Tregs contribute to tumor progression and the worse prognosis in RSCRC.

### The PLA2G4A/AA metabolic pathway was abnormally activated in RSCRC.

**Figure 4:**
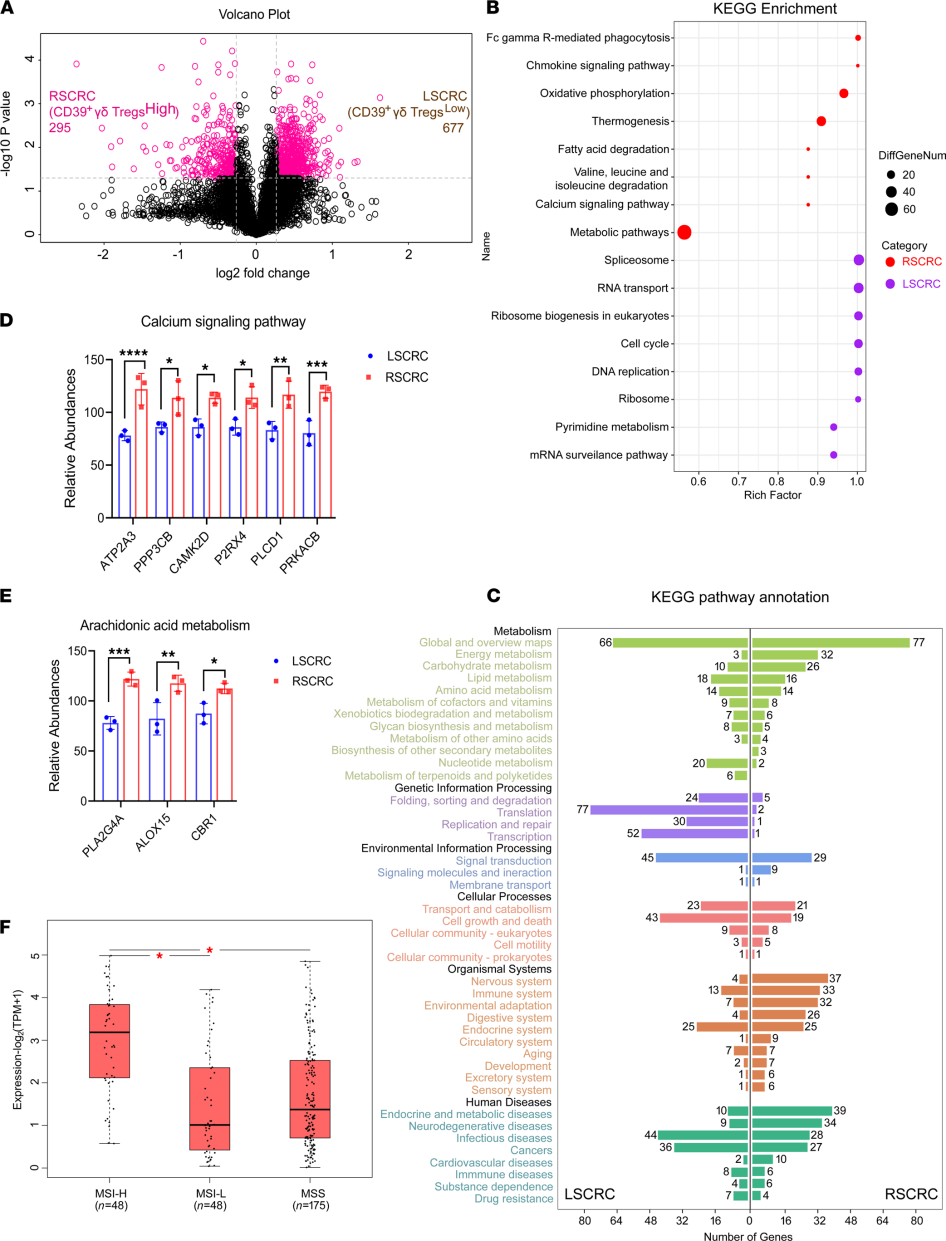
Difference of microenvironment between RSCRC and LSCRC. (**A**) The surgical specimens of RSCRC (*n* = 3) with high expression of CD39^+^γδ Tregs and the LSCRC (*n* = 3) with low expression of CD39^+^γδ Tregs detected by flow cytometry were then analyzed by tandem mass tag–based (TMT-based) quantitative proteomics. The profiling experiments resulted in quantification of 972 differential proteins — among them, 295 high expression proteins were found in RSCRC and 677 in LSCRC. A volcano plot indicating results from protein-level differential analysis comparing RSCRC with LSCRC samples. Dotted lines indicated that the cut-offs used to define regulated proteins (|log_2_ FC| > 1.2, adjusted *P* < 0.05). (**B**) A bubble chart displayed KEGG enrichment pathway of abnormal expression proteins in RSCRC/LSCRC. (**C**) Bar plot of secondary classification of KEGG pathway annotation highlighted features of RSCRC/LSCRC microenvironment. (**D** and **E**) The expression of calcium signaling pathway–related proteins (**D**) and arachidonic acid metabolism–related proteins (**E**) in RSCRC and LSCRC analyzed by quantitative proteomics. Data represent mean ± SEM, using 2-way ANOVA followed by Sidak’s multiple-comparisons test. (**F**) *PLA2G4A* mRNA expression in colorectal cancer with MSI-high/MSI-low or MSS based on data obtained from the GEPIA database. One-way ANOVA test was used. MSI, microsatellite instability; MSS, microsatellite stable. *P < 0.05; **P < 0.01; ***P < 0.001; ****P < 0.0001.

To explore the molecular mechanism leading to the differences of CD39^+^γδ Tregs derived from RSCRC and LSCRC, we collected fresh tissue samples of stage III–IV RSCRC with high expression of CD39^+^γδ Tregs and the LSCRC with low expression of CD39^+^γδ Tregs detected by flow cytometry, and we identified 972 differential proteins by liquid chromatography–mass spectrometry–based (LC-MS–based) quantitative proteomic analysis with tandem mass tag (TMT) (Figure 4A). By analyzing KEGG pathway enrichment, we found that the high expression proteins in the RSCRC microenvironment were mainly concentrated in the metabolic pathway (Figure 4B). In contrast, the abnormal expression proteins in the LSCRC microenvironment were primarily involved in cell proliferation–related DNA replication and repair, transcription, translation, and protein folding (Figure 4, B and C). According to these results, it is speculated that CD39^+^γδ Tregs^lo^ LSCRC has stronger local proliferation ability and is more sensitive to anticancer drugs targeting cell growth signaling pathway compared with CD39^+^γδ Tregs^hi^ RSCRC. Notably, since the NCCN guidelines in 2017 first proposed the difference in drug recommendation for RSCRC/LSCRC, NCCN guidelines for CRC diagnosis and treatment were updated again in 2019 (24). Cetuximab, an inhibitor of epidermal growth factor receptor, is no longer recommended for preoperative conversion therapy in patients with stage IV RSCRC (previously, the effect of cetuximab as the first-line treatment for patients with stage IV CRC has been confirmed, and some patients obtained surgery opportunity) (24–26). Clinical practice confirmed our results and conjectures. Besides, the proteins involved in the regulation of the immune system were predominantly expressed in RSCRC (RSCRC versus LSCRC = 33 versus 13, respectively; Figure 4C).

The considerable differences in the microenvironment of RSCRC and LSCRC raise an interesting question of how colorectal cells educate immune cells in the distinct microenvironment. By further analyzing the above quantitative proteomic results, we found that the calcium-dependent PLA2G4A/AA metabolic pathway was abnormally activated in RSCRC (Figure 4, D and E). Meanwhile, the abnormally activated function of FcγR-mediated phagocytosis in RSCRC (Figure 4B) is also dependent on the calcium pathway and PLA2/AA pathway (KEGG pathway: map04666). PLA2G4A is the most abundant subtype of cytosolic phospholipase A2 (cPLA2), which is a calcium-dependent protein. When cells are stimulated, specific transduction pathways regulate the increase of intracellular calcium concentration, and Ca^2+^ binds to the N-terminal C2 domain of cPLA2, which promotes the transfer of enzymes from the cytoplasm to the endomembrane (27). cPLA2 then hydrolyzes the acyl bond to produce AA and lysophosphatidylcholine. Subsequently, AA is converted into eicosanoid, prostaglandins, leukotrienes, thromboxane, and other signaling lipid metabolites that may regulate T cell differentiation and trigger inflammatory responses (27). In addition, we analyzed the GEPIA2 analysis data set and found that the expression levels of *PLA2G4A* in microsatellite instability–high (MSI-H) colorectal tumors were significantly higher than that in MSI-low (MSI-L) and microsatellite stable (MSS) tumors (Figure 4F; MSI-H, *n* = 48; MSI-L, *n* = 48; MSS, *n* = 175). These results indicate the value of PLA2G4A/AA metabolism in exploring the immune microenvironment difference between RSCRC and LSCRC.

### PLA2G4A induced the expression of CD39 on γδ-TILs.

**Figure 5:**
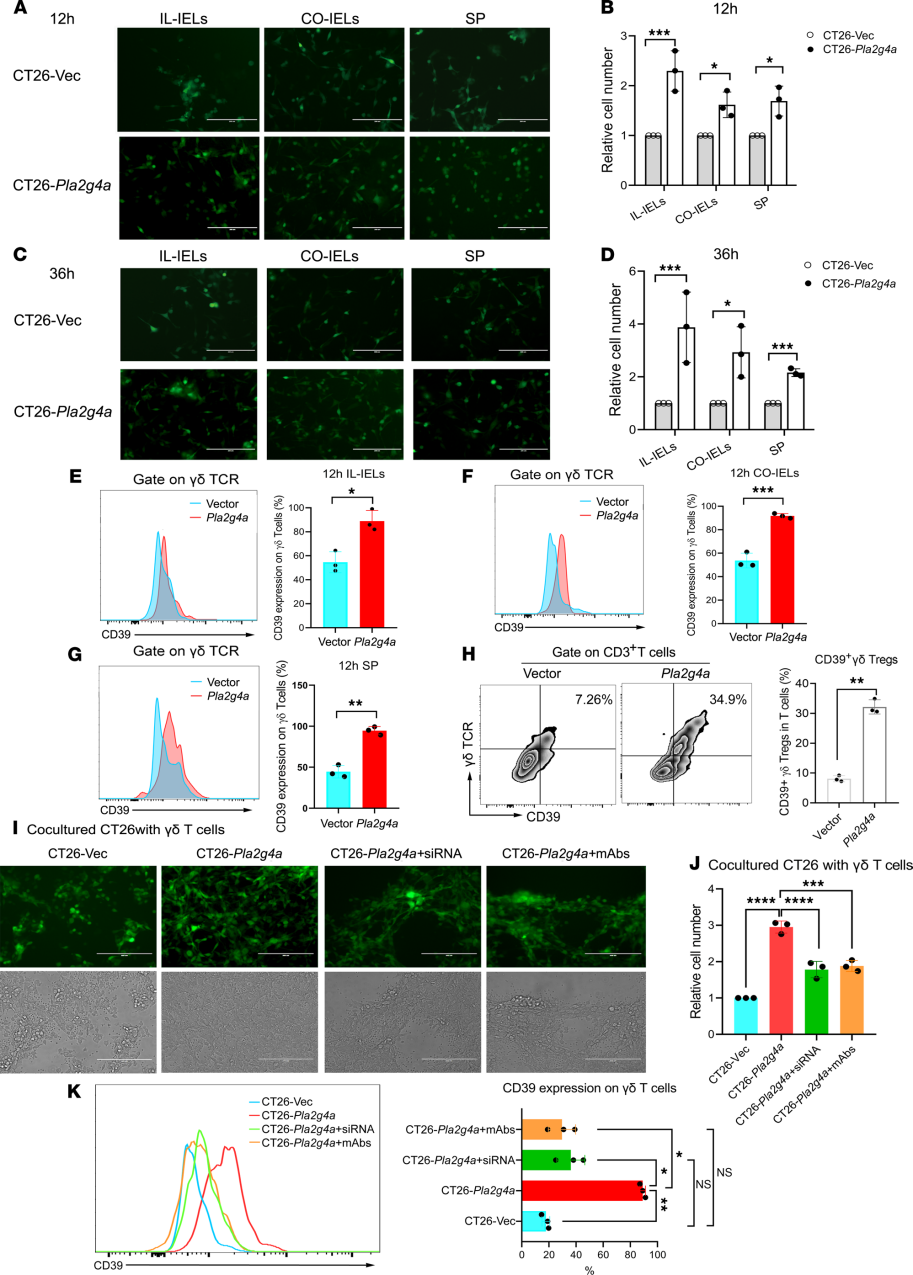
Overexpression of Pla2g4a in CT26-induced CD39+γδ Tregs in vitro. CT26-*Pla2g4a* and CT26-Vec cells were cocultured with intraepithelial lymphocytes derived from the ileum (IL-IELs), colon (CO-IELs), or spleen of BALB/c mice. (A and **C**) Representative pictures showed the number of CT26 cells in the coculture system after 12 (**A**) and 36 hours (**C**). Scale bars: 200 μm. (**B** and **D**) Bar diagrams summarizes the cell numbers of CT26-*Pla2g4a* and CT26-Vec cells cocultured with IL-IELs after 12 (**B**) and 36 (**D**) hours. Data are shown as mean ± SEM, using 2-way ANOVA followed by Sidak’s multiple-comparisons test. (**E–G**) Representative histogram plot and bar diagram summarizes the expression of CD39 on γδ T cells in IL-IELs (**E**), CO-IELs (**F**) and splenocytes (**G**) group. (**H**) Representative flow cytometric analysis of CD39^+^ Tregs in the total CD3^+^ T cells from splenocytes. Bar diagram summarizes the percentages of CD39^+^γδ Tregs. Unpaired 2-tailed *t* test was used to compare variables. (**I**) Silencing Pla2g4a in CT26-*Pla2g4a* rescued the antitumor response mediated by γδ T cells. CT26-*Pla2g4a* were transfected with *Pla2g4a* siRNA or control siRNA. After 24 hours, CT26-Vec, CT26-*Pla2g4a*, and CT26-*Pla2g4a*–knock-down cells were cocultured with γδ T cells sorted from BALB/c mice with or without anti-PLA2G4A antibody (5 mg/mL). Scale bars: 200 μm. (**J**) Bar diagram summarizes the cell number of CT26. (**K**) Representative histogram plot of CD39 expression on γδ T cells in each group by flow cytometry. Bar diagram summarizes the frequency of CD39^+^ γδ Tregs/γδ T cells. One-way ANOVA followed by Tukey’s multiple-comparison test was used for multiple-group analysis. All of the experiments were repeated 3 times; *n* = 3/group. *P < 0.05, **P < 0.01, ***P < 0.001, ****P < 0.0001.

To elucidate whether overexpression of PLA2G4A in cancer cells activates the adenosine pathway of γδ T cells, we overexpressed *Pla2g4a* in the CT26 cell line. The overexpression was verified using quantitative PCR (qPCR) and Western blot (Supplemental Figure 3, A–C). Firstly, we ruled out the effect of overexpression of *Pla2g4a* itself on the growth and metastasis of CT26 cells (Supplemental Figure 3, D–F). Then, CT26-*Pla2g4a* and CT26-vector (CT26-Vec) cells were cocultured with intraepithelial lymphocytes (IELs) derived from the ileum (IL-IELs), colon (CO-IELs), or spleen of BALB/c mice. We observed the status of cells in the coculture system by fluorescence microscope after 12 and 36 hours and found both IELs and splenocytes damage CT26-Vec to a great extent, while CT26-*Pla2g4a* inhibited the antitumor immune response (Figure 5, A–D). On the other hand, lymphocytes were harvested at 12 and 36 hours to detect the expression of CD39 on γδ T cells by flow cytometry. As expected, the overwhelming majority of γδ T cells cocultured with CT26-*Pla2g4a* were CD39 positive (Figure 5, E–G). Consequently, the frequency of CD39^+^ γδ Tregs increased 3.8-fold at 12 hours when compared with control (Figure 5H). Consistently, we observed similar results at 36 hours (Supplemental Figure 4, A–D).

Furthermore, we identified a decreased cell number of CT26-Vec in as little as 2 hours after cocultured, with γδ T cells sorted by magnetic beads (Figure 5, I and J). In comparison, overexpressed *Pla2g4a* in CT26 cells weakened the antitumor immune response mediated by γδ T cells and induced the expression of CD39. Moreover, silencing *Pla2g4a* in CT26-*Pla2g4a* caused a reduction in CD39 expression on γδ T cells and rescued their function (Figure 5, I–K). CD39^+^γδ Tregs induced by CT26-*Pla2g4a* predominately produced IL-17A (Supplemental Figure 4E).

**Figure 6:**
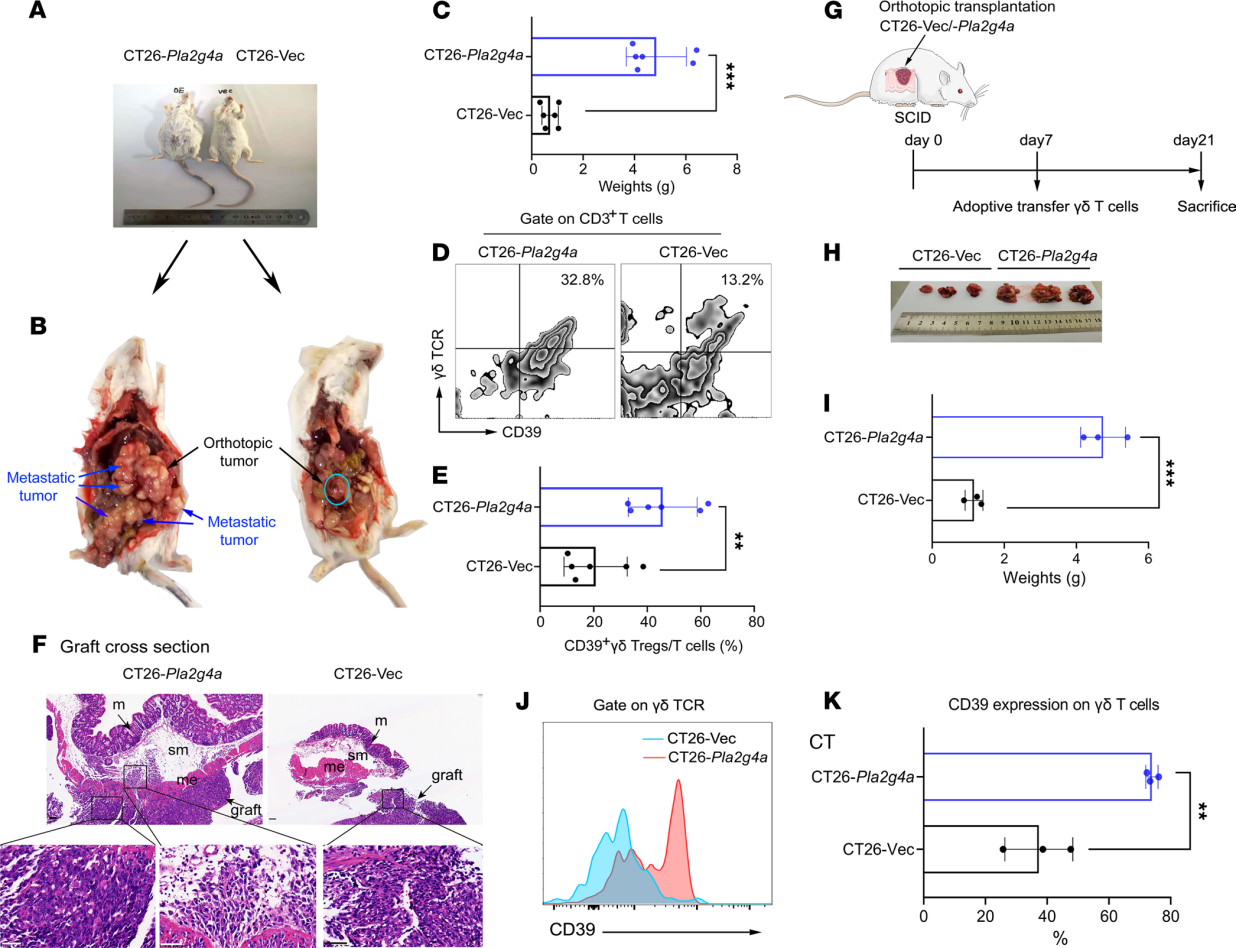
Overexpression of Pla2g4a in CT26 induced CD39+γδ Tregs using orthotopic murine models of colorectal cancer. (**A**) Approximately 3–4 weeks after CT26-*Pla2g4a* and CT26-Vec cells were surgically transplanted in the caecal submucosa of BALB/c mice, bloody ascites occurred, and the abdomen increased substantially in the CT26-*Pla2g4a* group. *n* = 6 per group. (**B**) The orthotopic tumor and metastatic tumor in the colon. (**C**) The tumor weight was robustly increased in the CT26-*Pla2g4a* group. (**D**) Representative flow cytometric analysis of CD39+γδ Tregs. (**E**) The frequency of CD39^+^γδ Tregs infiltrated in the orthotopic tumors in the 2 groups. (**F**) Representative H&E staining of graft cross-section in orthotopic tumor. (**G**) Schematic of SCID mice experimental design. *n* = 3 per group. (**H**) The tumor size was shown. (**I**) The weights of orthotopic tumors. (**J**) Representative histogram plot of CD39 expression on γδ T cells in each group by flow cytometry. (**K**) The frequency of CD39 positive cells. M, mucosa; me, muscolaris externa; sm, submucosa. Data shown are representative of 3 independent experiments. Data are shown as mean ± SEM. Unpaired 2-tailed *t* test was used to compare variables. **P < 0.01, ***P < 0.001. Scale bars: 50 μm.

To confirm the role of PlA2G4A^hi^ CRC on γδ T cells in vivo, we established an orthotopic murine model of CRC. As shown in Figure 6, A–C, *Pla2g4a* upregulation resulted in a substantial elevation of tumor weight and metastasis. All mice had bloody ascites in CT26-*Pla2g4a* groups. As expected, γδ-TILs isolated from the CT26-*Pla2g4a* group showed marked increases in the expression of CD39 when compared with control (Figure 6, D and E), which is consistent with the results of coculture in vitro. In addition, the results of H&E staining showed that the tumor invaded into the deep muscle layer (Figure 6F); on the other hand, it breaks through the serosal layer to achieve distal metastasis (Figure 6, B and F), indicating that upregulation of *Pla2g4a* promotes tumor invasion.

To eliminate the interference of αβ T cells, we constructed a SCID mouse CRC orthotopic transplantation adoptive transfer γδ T cell model (Figure 6G). Similarly, *Pla2g4a* upregulation promoted tumor progression (Figure 6, H and I) and induced the expression of CD39 on γδ-TILs (Figure 6, J and K).

These data collectively reveal a role for PLA2G4A^hi^ CRC in the activation of the CD39/adenosine pathway in γδ-TILs, thereby promoting tumor progression and metastasis.

### Exogenous AA induced CD39^+^γδ Treg polarization.

**Figure 7:**
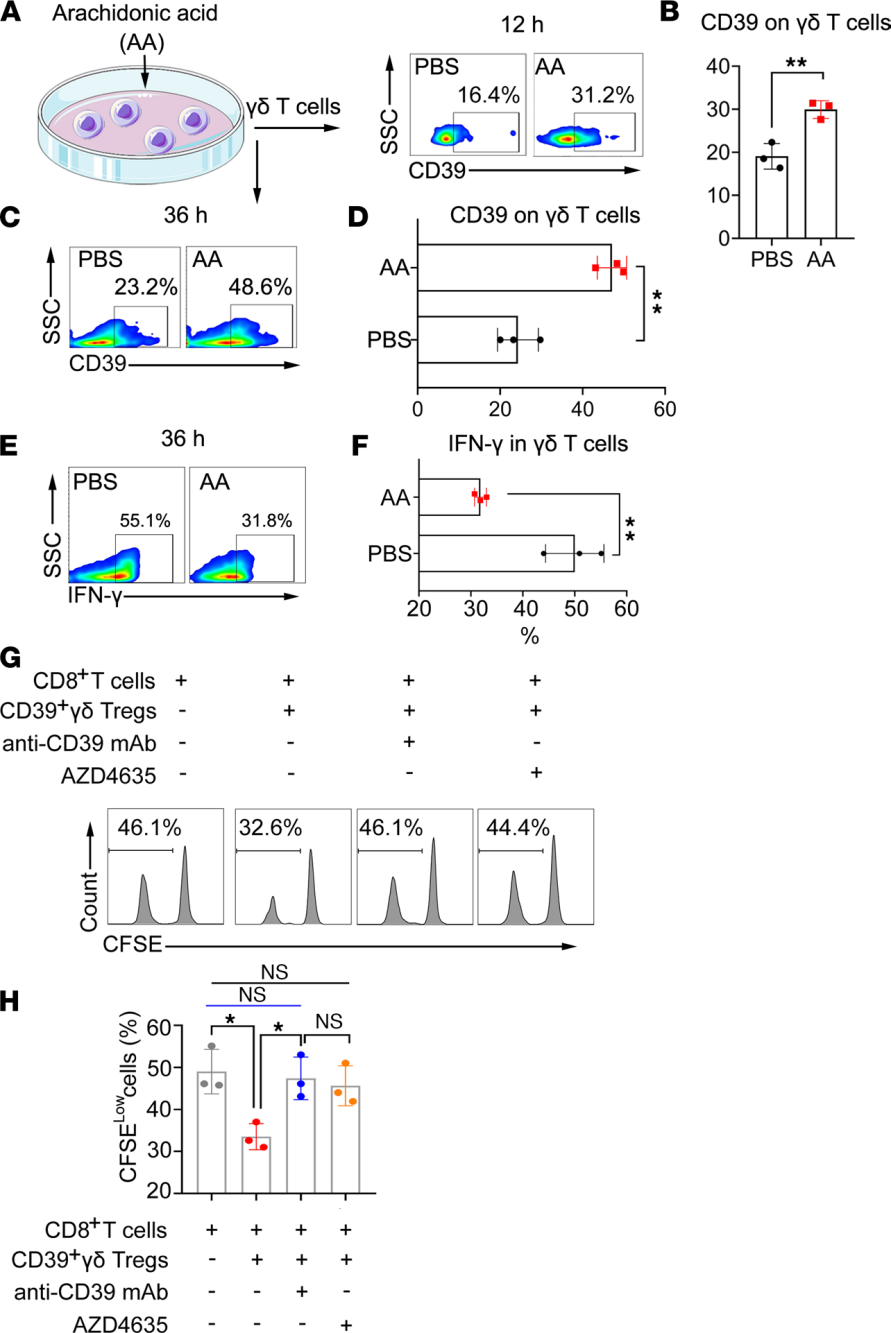
Exogenous AA promotes CD39+γδ Tregs polarization in vitro. (**A**–**D**) Exogenous AA (5 μM) induced the expression of CD39 on γδ T cells. γδ T cells sorted from BALB/c mice were treated with AA (5 μM) in vitro, and the expression of CD39 on γδ T cells was determined by flow cytometry at 12 hours (**A**) and 36 hours (**C**). The percentages of CD39 positive cells at 12 hours (**B**) and 36 hours (**D**) were calculated and compared using an unpaired *t* test (*n* = 3). (**E**) Exogenous AA (5 μM) decreased the production of IFN-γ in γδ T cells. The percentages of IFN-γ^+^ cells by intracellular staining at 36 hours (**F**) were calculated and compared using an unpaired *t* test (*n* = 3). (**G**) AA-induced CD39^+^γδ Tregs inhibited the proliferation of CD8^+^cells. AA-induced CD39^+^γδ Tregs sorted by flow cytometry were then mix with CFSE-labeled αβ T cells from BALB/c mice at a 1:5 ratio and activated by anti-CD3/CD28 mAb with or without anti-CD39 mAb/A2AR inhibitor AZD4635. CFSE was determined by flow cytometry, gating on CD8^+^cells. (**H**) Percentage of CFSE-low cells were calculated and compared using 1-way ANOVA followed by Tukey’s multiple-comparison test (*n* = 3). Data are represented as mean ± SEM of 2–3 independent experiments.

Because PLA2G4A catalyzes AA production from phospholipids, we further explored whether AA treatment could induce CD39^+^γδ Treg polarization in vitro. As shown in Figure 7, A–F, AA promoted the expression of CD39 on γδ T cells and decreased IFN-γ intracell production. Furthermore, we sorted the AA-induced CD39^+^γδ Tregs and determined the immunosuppressive function. Consistent with the results of RSCRC, AA-induced CD39^+^γδ Tregs significantly inhibited the proliferation of CD8^+^ T cells (Figure 7, G and H). Moreover, neutralization of CD39 by an antibody or blocking the high-affinity adenosine 2A receptor (A2AR) signaling rescued cell proliferation (Figure 7, G and H), confirming that CD39 adenosine leads to the immunosuppression of CD39^+^γδ Tregs.

### PLA2G4A shortened the survival of patients with RSCRC.

**Figure 8:**
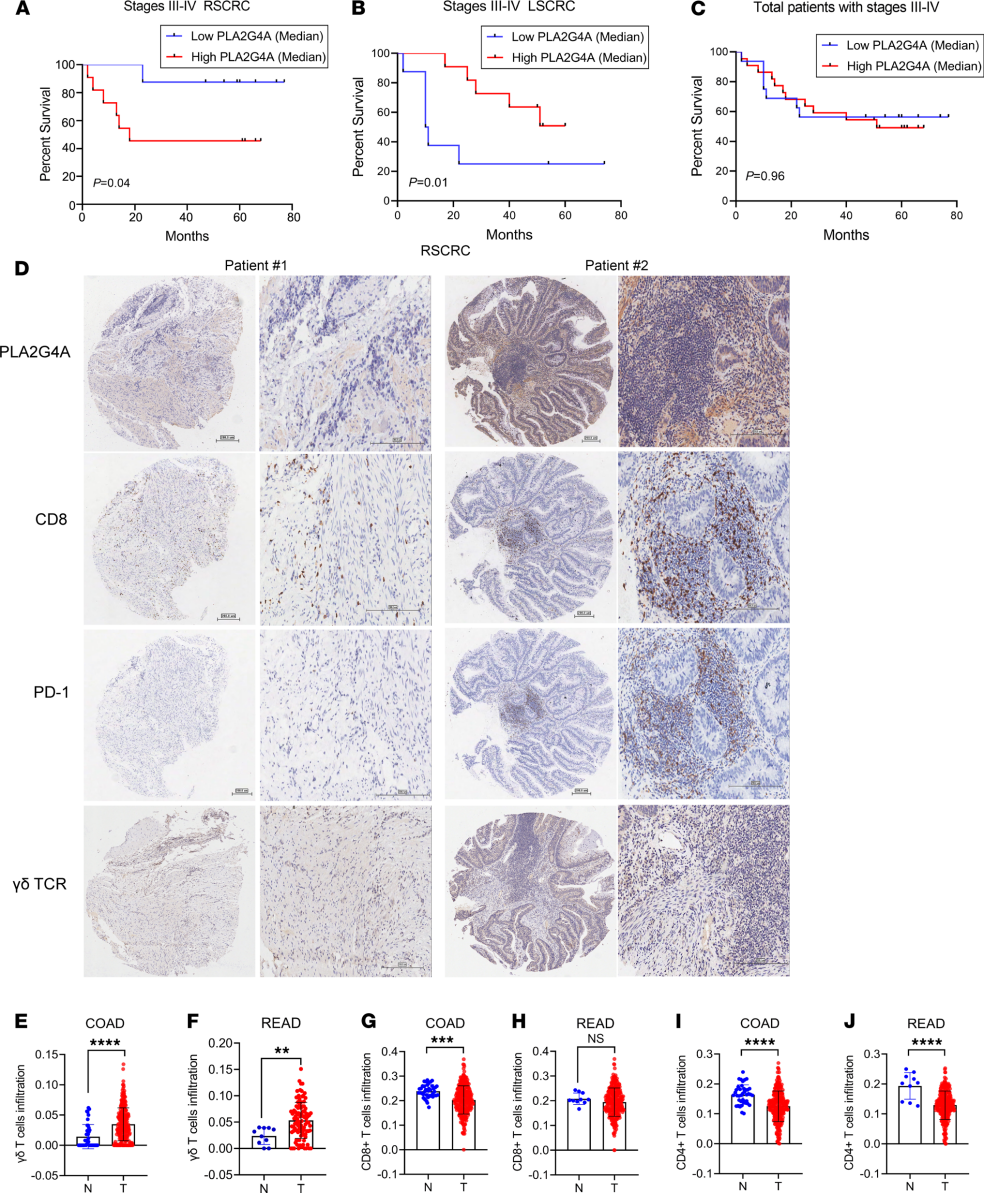
The relationship between PLA2G4A expression and the prognosis of patients with colorectal cancer. (**A**) Kaplan-Meier overall survival curves showed high-level expression of PLA2G4A correlated with poor prognosis in stage III–IV RSCRC. *n* = 20. (**B**) Patients with stage III–IV LSCRC with high-level of PLA2G4A had a better outcome in overall survival. *n* = 18. (**C**) Kaplan-Meier overall survival curves of patients with CRC. Log-rank test were used for analysis. *n* = 38. PLA2G4A median level was used as cut-off. (**D**) The representative IHC staining of PLA2G4A, CD8, PD-1, and γδ TCR expression in RSCRC samples (Patient 1 survival: 77 months, alive; Patient 2 survival: 14 months, deceased). Scale bars: 200 μm. (**E** and **F**) The infiltration of γδ T cells in COAD (**E**) and READ (**F**). (**G** and **H**) The infiltration of CD8^+^ T cells in COAD (**G**) and READ (**H**). (**I** and **J**) The infiltration of CD4^+^ T cells in COAD (**I**) and READ (**J**). COAD, *n* = 288, and normal, *n* = 40; READ, *n* = 95, and normal, *n* = 10. COAD, colon adenocarcinoma; READ, rectum adenocarcinoma. Data are shown as mean ± SEM. Unpaired 2-tailed *t* test was used to compare variables. ** *P* < 0.01, ***P < 0.001, ****P < 0.0001.

To evaluate the role of PLA2G4A in human CRC, we determined PLA2G4A expression by IHC in 94 patients with different histological grades of CRC. Interestingly, clinical survival analysis indicated that the patients with stage III–IV RSCRC with low PLA2G4A expression level had a better outcome in overall survival (*P* = 0.04) (Figure 8A), whereas a low-PLA2G4A expression indicated poor prognosis in LSCRC (*P* = 0.01) (Figure 8B). There was no noticeable difference in overall survival between the 2 groups without distinguishing the tumor site (Figure 8C). We also observed no difference in overall survival between the 2 groups in patients with stage I–IV CRC (Supplemental Figure 5).

Clinicopathological analysis showed that PLA2G4A expression was positively correlated with the expression of γδ TCR, CD8, and PD-1(γδ TCR, *R* = 0.704, *P* = 0.01; CD8, *R* = 0.503, *P* = 0.024; PD-1, *R* = 0.577, *P* = 0.008) in stage III–IV RSCRC. γδ TCR (*P* = 0.01), CD8 (*P* = 0.021), and PD-1 (*P* = 0.003) were upregulated in the PLA2G4A^hi^ cohort (Table 2 and Figure 8D). Similarly, the rate of PD-1 expression was significantly increased in stage I–IV RSCRC with high expression of PLA2G4A (*P* = 0.017; Table 3). Among the 34 cases of PD-1^–^ patients, 18 (52.9%) showed PLA2G4A^hi^ expression, whereas, among the 12 cases of PD-1 positive patients, 11 (91.7%) showed PLA2G4A^hi^ expression (Table 3). These results highlight the potential of PLA2G4A in predicting and evaluating the immune microenvironment of such patients. Consistently, we also observed increased infiltration of CD8^+^ T cells and elevated expression of PD-1 in the CT26-*Pla2g4a* mice (Supplemental Figure 6). We did not observe the correlation between PLA2G4A and PD-1 in LSCRC (Supplemental Table 1). Thus, these clinical data in human samples suggest that the expression of PLA2G4A alters in LSCRC/RSCRC.

In addition, we analyzed the ImmuCellAI analysis data set and found that the infiltration of γδ T cells in both colon adenocarcinoma (COAD) and rectum adenocarcinoma (READ) were significantly increased compared with normal tissues (Figure 8, E and F; COAD [*n* = 288], normal [*n* = 40]; READ [*n* = 95], normal [*n* = 10]), while CD4^+^ T cells were decreased (Figure 8, I and J). Compared with normal tissues, the infiltration of CD8^+^ T cells decreased in COAD (Figure 8G). There was no difference in CD8^+^ T cells between the tumor and normal tissues in the rectum (Figure 8H). These data implicate that γδ T cells may play more critical roles than αβ T cells in mediating the tumor-specific immune response in the human CRC microenvironment, highlighting that the transformation of γδ T cells into CD39^+^γδ Tregs promotes tumor immune escape.

Taken together, the above results reveal that RSCRC recruits γδ T cells and induces CD39^+^γδ Treg polarization through the PLA2G4A/AA pathway. On the one hand, the antitumor effect mediated by γδ-TIL is greatly reduced. On the other hand, CD39^+^γδ Tregs further weaken the immune function of αβ T cells via adenosine, thus promoting tumor progression.

## Discussion

In the present study, we aimed to define the distribution and function characteristics of CD39^+^γδ Tregs in human RSCRC and LSCRC. Besides the known differences in cancer cells themselves, our findings highlight the domestication of immune cells by distinct molecular subtypes of cancer; in return, tumor-educated immunosuppressive cells facilitate tumor progression. Specifically, we have demonstrated that the frequency of CD39^+^γδ-TILs is greatly increased in RSCRC, with abnormal activation of the PLA2G4A/AA pathway — along with upregulation of IL-17A and adenosine — and, consequently, enhanced immunosuppressive function. We discovered that high expression of PLA2G4A suggested poor prognosis in RSCRC. While for LSCRC, relatively “cold” tumors, the upregulation of PLA2G4A prolonged survival. These observations indicate the importance of studying the crosstalk between cancer cells and immune cells by distinguishing the primary tumor site and offering precise clinical treatment insights.

The critical problem that restricts immunotherapy application in CRC is that it is difficult for immune cells to enter the local tumor. We hope to make a breakthrough in immunotherapy of CRC by restoring the antitumor immune response of effector T cells resident in intestinal epithelial tissue. The γδ T cells in the colon are vital to the first line of defense in intestinal tissue immune surveillance (28). The γδ T cells in this organ are composed of resident and infiltrating cells. The resident subsets are called IELs. γδ IELs account for 30% and 50% of IELs in humans and mice, respectively (29, 30). γδ T cells can not only dissolve cancer cells directly through the perforin granzyme pathway (31, 32), but also interact with B cells, DC, αβ T cells, and NK cells to play an indirect antitumor effect (33). Compared with αβ T cells, γδ T cells are more likely to migrate into other tissues (33) and can quickly respond to targets and release effector cytokines, such as IFN-γ (34). More importantly, γδ T cells have higher invasive ability and function in the tumor hypoxia environment (32). Therefore, γδ T cells are advantageous candidate cells in tumor immunotherapy. However, recent researches have demonstrated that tumor-educated γδ T cells could function in suppressing antitumor immunity. A previous study indicates that the γδ T cells could be domesticated by CRC and polarize to CD39^+^γδ Tregs and could play an immunosuppressive role by secreting adenosine (14). We also observed the above phenomenon here, but the primary tumor site on cell interaction in the microenvironment has not been elucidated. Here, our data show a higher accumulation of tumor-infiltrating CD39^+^γδ Tregs in RSCRC than paired normal tissue and LSCRC, along with increased production of IL-17A and adenosine, as well as upregulation of Tim-3. By comparing T cells in fresh tissues of patients with double primary CRC, we found that CD39^+^γδ Tregs from the RSCRC exert stronger immunosuppressive ability than LSCRC.

In order to apply the γδ T cell in the immunotherapy of solid tumors, it is necessary to prevent γδ T cells from being transformed from antitumor to protumor after entering the tumor microenvironment. Therefore, it is urgent to study the pathway of tumor-inducing γδ Tregs thoroughly. Despite a large focus of tumor-educated γδ Treg research centered on TGF-β and its related signaling pathway (14, 35–37), we did not find the difference between the TGF-β pathway with CD39^+^γδ Tregs^lo^ CRC compared with CD39^+^γδ Tregs^hi^ CRC by proteomic analysis. We further found that the PLA2G4A/AA pathway was abnormally activated in RSCRC with high expression of CD39^+^γδ Tregs. Notably, this pathway is involved in a number of abnormal activation signaling pathways and biological functions of RSCRC. The involvement of the PLA2G4A/AA pathway in the inflammatory process and T cell polarization has been confirmed by many studies (38–41). Prostaglandin E2 (PGE2) produced by cyclooxygenase-catalyzing (COX-catalyzing) AA can induce Th17 production in intestinal mucosa by enhancing IL-23 signal (38, 39), while PGE2 can induce CD4^+^Foxp3^+^ T cells in mesenteric lymph nodes (40). ALOX15 catalyzes AA to 15s-HpETE, which in turn promotes the transformation of immature CD4^+^ T cells into T helper follicular cells (41). Therefore, we further studied and confirmed that overexpression of PLA2G4A induced the activation of the CD39/adenosine pathway in γδ T cell to impair its antitumor response, consequently promoting tumor progression. Besides, a large amount of PGE2 released by pancreatic ductal adenocarcinoma cells with upregulation of COX2 impairs γδ T cell cytotoxicity (42). However, we did not discover a difference in COX2 expression in CRC with CD39^+^γδ Tregs^lo^ and CD39^+^γδ Tregs^hi^. Interestingly, 15-LOX increased in the latter (Figure 3). Therefore, we speculate that CRC with PLA2G4A/AA abnormal activation may induce CD39^+^γδ Tregs through the downstream lipoxygenase pathway. Further exploration is needed to confirm the above hypothesis. Moreover, it is also possible that the cancer cells secrete more AA to the intercellular substance, and metabolites of AA in the γδ T cells induce the expression of CD39. We initially explored this possibility and found that γδ T cells treated with AA in vitro showed increased CD39 expression, decreased intracellular IFN-γ, and the ability to inhibit the proliferation of CD8^+^ T cells (Figure 6). More research is needed to determine the best methods.

Notably, γδ-TILs from Patients with CRC were a dominantly tissue-resident Vδ1 subset rather than a circulating Vδ2 subset (Supplemental Figure 1). Therefore, instead of simply constructing a s.c. tumor model, we inoculated CT26 cells into the intestinal wall of mice to mimic the effect of CRC on the intestinal-resident γδ T cells. Although human and mouse γδ T cell subsets are not homologous, they are both intestinal resident γδ T cells, and the induction effects of tumors on these cells are similar. To explore the interaction between CRC and tissue-resident γδ T cells, the mouse models used in this study are currently a relatively feasible and appropriate solution.

Previous studies have suggested that the left- and right-sided large intestine have different immune environments, leading to the distinct immune responses to stimulation (43). The size and number of submucosal isolated lymphoid follicles (SM-ILFs), which comprise a central follicle of B cells surrounded by T cells, in the right large intestine are larger than those in the left large intestine. Interestingly, SM-ILFs are the main adaptive immune–inductive sites of the cecum and proximal and transverse colon (right-sided), whereas mucosal isolated lymphoid follicles (M-ILFs) serve such functions in the ileum and distal colon (left-sided) (43). Based on the biology of the disease, consensus molecular subtypes (CMSs) divides CRCs into 1 of 4 CMS groups: CMS1, MSI immune; CMS2, canonical; CMS3, metabolic; and CMS4, mesenchymal (44). CMS1 is mainly RSCRC, while CMS1 proportion in LSCRC is only 9.01% (31 of 344) (45, 46). According to the above mechanism study results and clinical studies, we speculate that the right-sided large intestine may have more abundant immune cells. When the level of PLA2G4A increases, CD39^+^γδ Tregs would be induced to form an immunosuppressive microenvironment and promote tumor progression (the present study provided relevant evidence). However, gut-associated lymphoid tissues (GALTs) of the left-sided large intestine are not rich enough. The inflammatory response mediated by the PLA2G4A/AA signaling pathway may help recruit immune cells, thus enhancing the immune response. This may be why the expression of PLA2G4A has different effects on the survival of patients with RSCRC/LSCRC. In addition, according to our results of flow cytometry and proteomics, the CD39^+^γδ Tregs^lo^ LSCRC belongs to CMS2, while CD39^+^γδ Tregs^hi^ RSCRC is similar to CMS1. There is a great difference in the domestication of γδ T cells by the 2 types of tumors, which is certainly not fully explained by 1 pathway. From the perspective of immune cells, the function of Tregs needs the continuous stimulation and maintenance of various immunosuppressive cytokines and small molecule metabolites. Our results show that the adenosine level in RSCRC was significantly higher than that of LSCRC, suggesting that the microenvironment of different sites affected the immune regulation function of CD39^+^γδ Tregs. All of these factors may contribute to the difference in overall survival. Further studies and larger samples are needed to confirm this hypothesis.

In summary, the present study indicates that the RSCRC microenvironment with abnormal activation of PLA2G4A educated γδ T cells into CD39^+^ γδ Tregs, which produce the protumor cytokine IL-17A and adenosine, consequently establishing a potent immunosuppressive microenvironment for promoting immune evasion and tumor metastasis. Our work revealed CD39^+^ γδ Tregs as a critical factor correlated with the progression of PLA2G4A^hi^ RSCRC and deepen the understanding of tumor microenvironment and immunosuppression.

## Methods

### Patient tissue samples.

A total of 129 patients with CRC who were admitted and treated at Tianjin Medical University Cancer Institute and Hospital between February 2014 and September 2020 were enrolled randomly. During surgical resection, specimen (both tumor and paired normal tissues) were obtained from the treatment-naive patients with CRC. The clinical features of the patients are presented in Table 1. The records of all of the patients contained basic information, including tumor-node-metastasis (TNM) stage, the degree of differentiation, lymph node metastasis, and distant metastasis, according to the 2002 International Cancer Alliance TNM staging criteria (47).

### Isolation of tissue-infiltrating cells.

Fresh colorectal tumor tissues and paired normal tissues from patients with CRC were prepared by mechanical disruption, followed by digestion with 0.5 mg/mL collagenase type IV (catalog C5138; Sigma-Aldrich) in 10% FBS with 10 U/mL DNase I in RPMI-1640 medium (both from Thermo Fisher Scientific) at 37°C for 30 minutes. Digested tissues were incubated for 5 minutes at 37°C with EDTA (0.5M) to prevent DC/T cell aggregation and filtered through a 100 μm and a 40 μm filter. The isolation and culture of tissue/tumor-infiltrating cells were performed as previously described (48).

For CD39^+^γδ Treg functional experiments, single-cell suspensions from tissues were generated rapidly and gently using the Tumor Dissociation Kit (catalog 130-095-929; Miltenyi Biotec) and gentleMACS Octo Dissociator (catalog 130-095-937; Miltenyi Biotec).

### Flow cytometric analysis.

Tumor/paired normal tissue-infiltrating cells (1 × 10^6^ cells) were incubated with human PerCP anti-CD45 mAb (1:20; catalog 304026; BioLegend), FITC anti-CD3 mAb (1:20; catalog 300452; BioLegend), APC anti-CD4 mAb (1:20; catalog 357408; BioLegend), APC anti-CD8 mAb (1:20; catalog 344722; BioLegend), APC anti–TCR γ/δ mAb (1:20; catalog 331212; BioLegend), FITC anti–TCR Vδ1 mAb (1:25; GTX79223; GeneTex), PE anti–human TCR Vδ2 (1:20; catalog 331408; BioLegend), PE anti-CD39 mAb (1:20; catalog 328208; BioLegend), APC/Cy7 anti–PD-1 mAb (1:20; catalog 329921; BioLegend), and PE/Cy7 anti–Tim-3 mAb (1:20; catalog 345013; BioLegend) in cell staining buffer (catalog 420201; BioLegend) for 15 minutes at room temperature (RT). For intracellular staining, cells were incubated with PE/Cy7 anti–IFN-γ mAb (1:20; catalog 502527; BioLegend) and APC/Cy7 anti–IL-17A mAb (1:20; catalog 512334; BioLegend) in 100 μL intracellular staining permeabilization buffer (catalog 421002; BioLegend) for 15 minutes at RT. LIVE/DEAD Fixable Dead Cell Stain Kits (catalog L34963; Invitrogen) were used to stain dead cells. After washing with PBS and centrifugation at 400*g* for 5 min at 4°C, 1 × 10^6^ cells were suspended in 300 μL cell staining buffer (catalog 420201; BioLegend) and analyzed on a BD FACSCalibur (BD Biosciences) flow cytometer. The data were analyzed using FlowJo v10 software (FlowJo).

For mouse experiment, FITC anti-CD3 mAb (1:20; catalog 100204; BioLegend), APC anti–TCR γ/δ mAb (1:20; catalog 118116; BioLegend), PE anti-CD39 mAb (1:20; catalog 143804; BioLegend), APC anti-CD8a mAb (1:20; catalog 100712; BioLegend), APC anti-CD4 mAb (1:20; catalog 100412; BioLegend), PE/Cy7 anti–IFN-γ mAb (1:20; catalog 505826; BioLegend), and APC/Cy7 anti–IL-17A mAb (1:20; catalog 506940; BioLegend) were used for staining and detected by flow cytometry.

### Extracellular adenosine detection.

Adenosine concentrations were measured using a HPLC system equipped with an Inertsil ODS-SP C18 chromatogram column (4.6 mm × 250 mm, 5 mm) using a mobile phase consisting of methanol and 0.05M potassium dihydrogen phosphate at the volume ratio of 10:90. Identification and quantification of adenosine peaks were done by comparison to retention times of known standards (catalog 123179-97-5; Selleck) and peak integration and normalization.

### In vitro suppressive assay.

For the CD39^+^γδ Treg–mediated CD4^+^/CD8^+^ T cell suppression experiment, CD39^+^ γδ Tregs were sorted from tumors and paired normal tissues of patients with RSCRC/LSCRC and then were cocultured with allogeneic peripheral blood CFSE-labeled (5 μM; catalog 65-0850-84; Invitrogen) CD4^+^/CD8^+^ T cells at a 1:5 ratio in the presence of ImmunoCult Human CD3/CD28 T Cell Activator (catalog 10971; Stemcell Technologies) for 5 days. An anti-CD39 mAb (5 μg/mL; ab178572; Abcam) and an A2AR inhibitor, AZD4635 (5 μM; M7531; AbMole), were used for rescue experiments. CD4^+^/CD8^+^ T cells were separated by RosetteSep Human CD4^+^ T Cells Enrichment Cocktail (catalog 15062; Stemcell Technologies) and an EasySep Direct Human CD8^+^ T Cell Isolation Kit (catalog 19663; Stemcell Technologies). On day 5, cells were harvested and intracellularly stained with anti–IFN-γ mAb. CFSE-low cells and IFN-γ production were detected by flow cytometry. Peripheral blood samples of the healthy donor were collected in Tianjin Medical University Cancer Institute and Hospital.

### TMT proteome analysis.

We selected 6 samples of CD39^+^ γδ Tregs^hi^ RSCRC and CD39^+^ γδ Tregs^lo^ LSCRC for proteomic characterization using TMT (Thermo Fisher Scientific). After protein extraction, the whole protein in tissue cells was obtained. The whole protein was hydrolyzed into peptides by trypsin, and all peptides were labeled by TMT reagent. The labeled peptides of each group were mixed and graded by Agilent 1260 infinity II HPLC system. Buffer A was 10 mm HCOONH_4_, 5% ACN, pH 10.0, and buffer B was 10 mm HCOONH_4_, 85% ACN, pH 10.0. The chromatographic column was equilibrated with buffer A. Samples were loaded into the chromatographic column by the manual automatic injector for separation. The flow rate was 1 mL/min. The eluting components were lyophilized and then were redissolved with 0.1% FA. Each sample was separated by Easy nLC system (Thermo Fisher Scientific) and then analyzed by Q Exactive plus mass spectrometer (Thermo Fisher Scientific).

Proteome discoverer 2.1 (Thermo Fisher Scientific) software transformed the original atlas file generated by Q Exactive plus into a .mgf file, which was submitted to the MASCOT2.6 server for database retrieval through the built-in tool of the software. Then the database search file (.dat file) formed on the MASCOT server was sent back to the software through the proteome discoverer 2.1, and the data were filtered according to the FDR < 0.01 standard to obtain highly reliable qualitative results. The database used in this study was Uniprot_HomoSapiens_20386_20180905. The mass spectrometry proteomics data have been deposited to the ProteomeXchange Consortium via the PRIDE partner repository with the dataset identifier PXD027323.

### Analysis of public data sets.

RNA sequencing–based gene expression data in CRC was obtained from the Gene Expression Profiling Interactive Analysis (GEPIA) database (49) and ImmuCellAI analysis data set (50) for cancer genomics. For gene expression, *P* < 0.01 was used as the cut-off.

### Lentivirus construction and stable cell line establishment.

The mouse *Pla2g4a* gene was amplified by PCR with primers 5′-AGGTCGACTCTAGAGGATCCCGCCACCATGTCTTTCATAGATCCTTATCAGCAC-3′ (forward) and 5′-TCCTTGTAGTCCATACCCACAGTGGGTTTACTTAGAAACTC-3′ (reverse). Next, the PCR products were inserted into the BamH I and Agel sites of GV492 vector (Genechem Co.). Lentiviruses were produced by 293T cells for stable transfection of cell lines, following the manufacturer’s instructions. The empty vector was transfected into the cells as a control. A total of 1 × 10^5^ CT26 cells in 1 mL 1640 medium with 4 μg/mL polybrene were infected with 2 × 10^6^ TU/mL lentivirus. After 48 hours, 3 μg/mL puromycin was added for selection. The *Pla2g4a* overexpression cells (CT26-*Pla2g4a*) and control cells (CT26-Vec) were observed and photographed by fluorescence microscope (Supplemental Figure 3A). Transfection efficiency was determined by real-time PCR and Western blot (Supplemental Figure 3, B and C). CT26 and 293T cells were purchased from American Type Culture Collection (ATCC).

### RNA isolation and quantitative PCR (qPCR).

Total RNA was extracted from cell lines using an RNApure Tissue & Cell Kit (Cwbiotech) following the manufacturer’s protocol. qPCR for mRNAs was performed as described previously with primers *Pla2g4a* 5′-AAAAGAGTACCAAAGCGACAAC-3′ (forward) and 5′-CGTCCTTCTCGGGTATTGAATA-3′(reverse) (51). Commercial mouse *Gapdh* (catalog B661304, Sangon Biotech) was used as the internal control.

### siRNA and transfection.

Small interfering RNA (siRNA) was used for transient knockdown of *Pla2g4a*. *Pla2g4a* siRNA (catalog sc-35098) and control siRNA (catalog sc-37007) were purchased from the Santa Cruz Biotechnology Inc. and used at a final concentration of 100 nmol/L. The cells were transfected using siRNA transfection reagent (catalog sc-29526) according to the manufacturer’s instructions.

### Isolation of IELs.

The mouse ileum and colon were separated, and Peyer’s Patches and the intestinal contents were removed with PBS. The isolated intestine was placed into cold RPMI-1640 containing 10% FBS to maximize cell viability. The intestine was cut open and then cut into 1 cm segments. The segments were incubated in 30 mL RPMI containing 93 μL 5% (w/v) dithiothreitol (DTT) + 60 μL 0.5 M EDTA + 500 μL FBS; they were stirred at 500 rpm for 15 minutes at 37°C. The digested tissue was filtered through a 100 μm cell strainer. In total, 20 mL of RPMI-1640 containing 10% FBS was used to rinse the strainer. The cells were collected and suspended with 3 mL 40% Percoll solution and then were gently superimposed on 2 mL 70% Percoll solution. IELs were collected after centrifuging at 600*g* for 20 minutes.

### In vitro CD39^+^ γδ Tregs induction by cancer cells.

IELs derived from the ileum (IL-IELs), colon (CO-IELs), or spleen of BALB/c mice were cocultured with CT26-Vec or CT26-*Pla2g4a* for 12 and 36 hours (IELs/CT26 = 1:4). For rescue experiments, γδ T cells sorted from IELs of BALB/c mice were cocultured with CT26-Vec or CT26-*Pla2g4a* (γδ T/CT26 = 1:1) with siRNA pretreatment for 2 hours using a mouse TCR γ/δ T cell Isolation Kit (catalog 130-092-125; Miltenyi Biotec). An anti-Pla2g4a antibody (5 μg/mL; catalog 110218; GeneTex) was used to neutralize PLA2G4A in cell culture supernatant. And then cells were washed, harvested, and then stained with PE anti-CD39 mAb (1:20; catalog 143804; BioLegend) and detected by flow cytometry.

### Orthotopic murine model of CRC.

Six- to 8-week-old male BALB/c mice or SCID mice were purchased from SPF Biotechnology Co. Ltd. and used for animal studies. CT26-Vec/*Pla2g4a* cells (1 × 10^7^) were suspended in 100 μL PBS containing 33 μL Matrigel (catalog 354234, Corning); then, cells were surgically transplanted into the submucosa of the caecal wall of a BALB/c mouse or a SCID mouse. The cecum was exteriorized through a midline abdominal incision, and cells was surgically transplanted in the caecal submucosa. Matrigel keeps the cells in place, allowing them to engraft and expand in the cecal wall. Over time, matrigel is degraded and the engrafted CT26 cells remains. Approximately 3–4 weeks after transplantation, the presence of primary tumors could be detected by abdominal palpation. Then, the BALB/c mice were sacrificed and the orthotopic tumor, intestinal metastasis, and distal organ metastasis were obtained. For SCID mice experiments, γδ T cells (5 × 10^6^)derived from BALB/c mice were adoptively transferred into a SCID mouse 7 days after orthotopic transplantation. Two weeks after adoptive transfer, the SCID mice were sacrificed.

### In vitro CD39^+^ γδ Tregs induction by AA.

Naive γδ T cells sorted from splenocytes of BALB/c mice were treated with AA (5 μM, catalog A5837; Sigma-Aldrich) using a mouse TCR γ/δ T cell Isolation Kit (Miltenyi Biotec) for 12 and 36 hours. Then, cells were washed, harvested, and stained with PE anti-CD39 mAb or intracellular stained with PE-Cy7 anti–IFN-γ mAb (1:20; catalog 505826; BioLegend) and detected by flow cytometry.

For the AA-induced CD39^+^γδ Treg–mediated CD8^+^ T cell suppression experiment, CD39^+^ γδ Tregs were sorted from above γδ T cells pretreated with AA (5 μM) and were then cocultured with αβ T cells labeled with CFSE (Invitrogen) at a 1:5 ratio for 2 days. αβ T cells were unlabeled by magnetic beads in the process of sorting γδ T cells. An anti-CD39 mAb (5 μg/mL; ab227840; Abcam) and an A2AR inhibitor, AZD4635 (5 μM; M7531; AbMole), were used for rescue experiments. Then, cells were washed, harvested, and stained with APC anti-CD8 mAb (1:20; catalog 100712; BioLegend). CFSE-low CD8^+^ T cells were detected by flow cytometry.

### IHC.

Colorectal tumor tissues and normal control tissues were fixed with 4% formaldehyde for 24 hours at RT, embedded in paraffin, and cut into 4 μm sections. The sections were dewaxed with xylene and then hydrated through a graded series of ethanol for 5 minutes in each percentage at RT. Slides were boiled in 10 mM sodium citrate buffer (pH 6.0) at 95°C for 10 minutes and subsequently incubated in 3% hydrogen peroxide for 10 minutes. Sections were blocked with 100–400 μL 5% normal goat serum in TBS-Tween (catalog 5425; Cell Signaling Technology) for 1 hour at RT. To assess PLA2G4A/CD8/PD-1/γδ TCR expression in the CRC specimens, sections were incubated with an anti-PLA2G4A antibody (dilution, 1:100; catalog sc-454; Santa Cruz Biotechnology Inc.), an anti-CD8 antibody (dilution, 1:200; catalog 85336; Cell Signaling Technology), an anti–PD-1 antibody (dilution, 1:200; catalog 86163; Cell Signaling Technology), or an anti–γδ TCR antibody (dilution, 1:100; catalog 331202; BioLegend) overnight at 4°C; they were then incubated with a horseradish peroxidase–conjugated secondary antibody (dilution, 1:1,000; catalog ab6721; Abcam) at RT for 1 hour. Following washing with PBS, the sections were incubated for 30 minutes at RT with streptavidin-biotin conjugated with horseradish peroxidase (dilution, 1:100; catalog KIT-0305; UltraSensitive SP [Mouse/Rabbit] IHC kit). Subsequently, the slides were stained with 3,3′-diaminobenzidine (Fuzhou Maixin Biotech Co. Ltd.), and the nuclei were counterstained with haematoxylin for 5 minutes at RT. Morphometric analyses of the tumor and paired normal tissues were performed using an Olympus BX51 light microscope (magnification, ×10,000; Olympus Corporation). Images were obtained from 10 randomly selected areas.

Staining score was calculated by multiplying percentage of positive cells with staining and intensity of staining. Staining intensity was scored as follows: 0, negative; 1, weakly positive; and 2, positive. According to the percentage of cancer cells per the whole tumor area: 0 (0%), 1 (<25%), 2 (26%–50%), 3 (51%–75%), and 4 (>75%). Samples were subsequently grouped into low-expression and high-expression groups. The median level was used as a cut-off.

### Statistics.

Statistical analysis was performed using GraphPad Prism v8 software (GraphPad Software Inc.). Data are presented as the mean ± SEM of 3 repeats. A χ^2^ test was used to evaluate the association between PLA2G4A expression and the clinicopathological parameters. A log-rank test was used to compare the survival curves of 2 groups. Two-way ANOVA followed by Sidak’s multiple-comparison test was used for multiple-group analyses. A 2-tailed unpaired Student’s *t* test was used for 2-group comparison. Pearson’s correlation analysis was used to analyze the correlation between the expression of PLA2G4A and the pathological grade or TNM stage. *P* < 0.05 was considered to indicate a statistically significant difference.

### Study approval.

All the samples were collected according to standard local protocols and after obtaining informed consent. The Ethics Committee from Tianjin Medical University Cancer Institute and Hospital provided approval for the study. The procedures for the handling and care of the mice were approved by the Animal Experimentation Ethics Committee of Tianjin Medical University Cancer Institute and Hospital.

## Author contributions

DK and YZ designed research studies. DK and TZ provided financial support and supervised the study. YZ conducted experiments, acquired data, and wrote the manuscript. LZ, DH, JW, and KL assisted with the collection and treatment of surgical specimens. JL and JG analyzed the data. All authors participated in the interpretation of data and editing of the manuscript for intellectual content. All authors approved the final manuscript.

## Supplementary Material

Supplemental data

## Figures and Tables

**Table 3:**
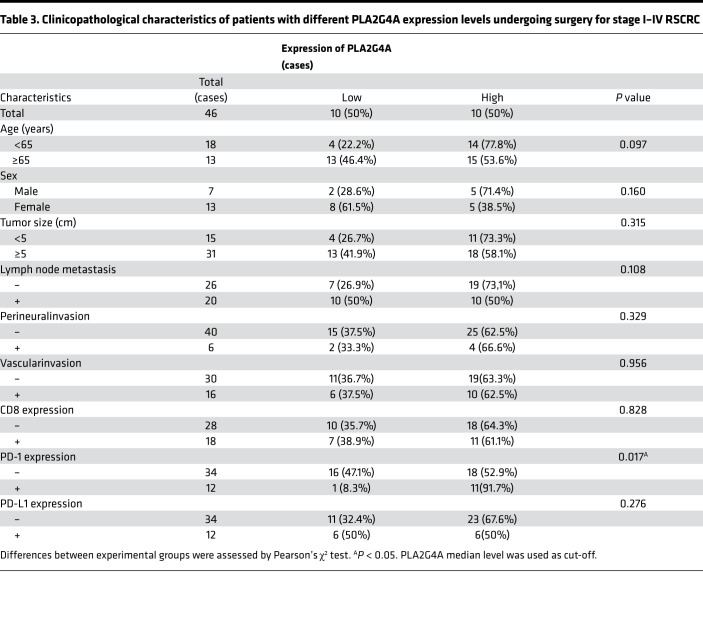
Clinicopathological characteristics of patients with different PLA2G4A expression levels undergoing surgery for stage I–IV RSCRC

**Table 2:**
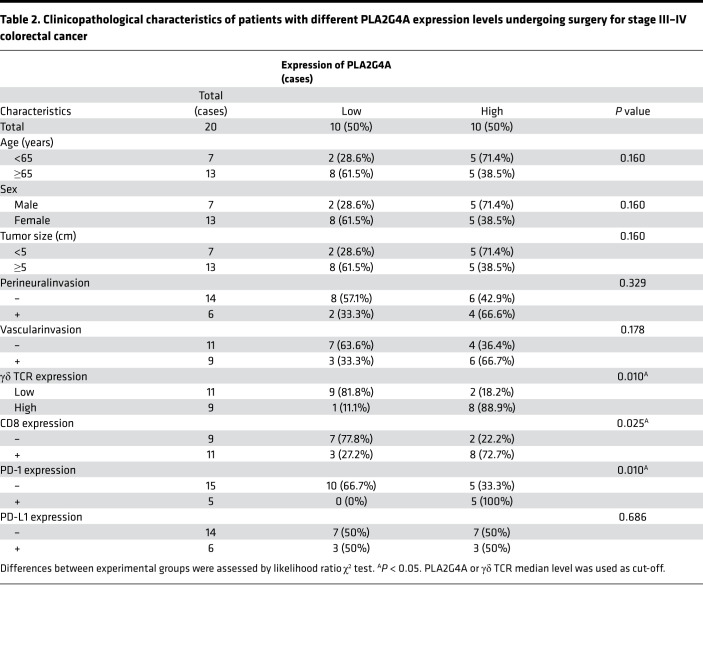
Clinicopathological characteristics of patients with different PLA2G4A expression levels undergoing surgery for stage III–IV colorectal cancer

**Table 1:**
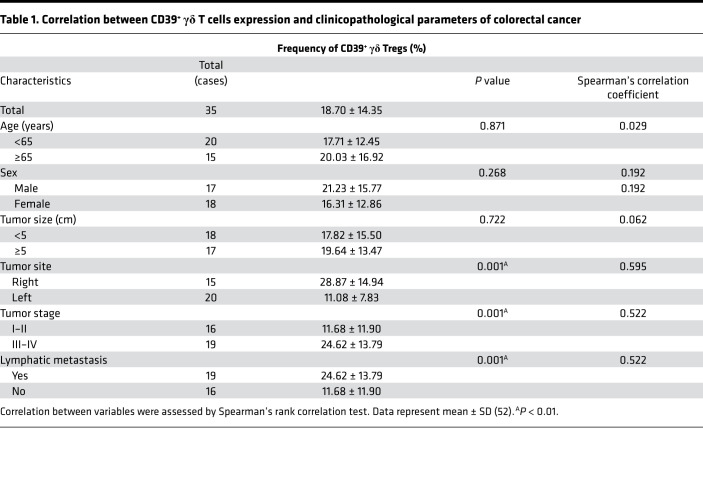
Correlation between CD39^+^ γδ T cells expression and clinicopathological parameters of colorectal cancer

## Version 1 07/20/2021

In-Press Preview

## Version 2 08/23/2021

Electronic publication
