# Supplementary material for: PLA2G4A promotes right-sided colorectal cancer progression by inducing CD39+γδ Treg polarization
Source: JCI Insight. 2021 Aug 23;6(16):e148028. doi: 10.1172/jci.insight.148028 (PMC8409991; doi:10.1172/jci.insight.148028)
Supplement: Supplemental data [file jciinsight-6-148028-s166.pdf]

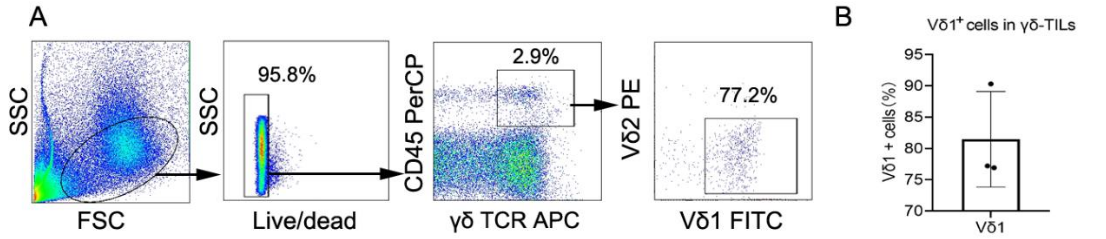

Supplementary Figure 1. The γδ-TILs were dominantly tissue-resident Vδ1 subset. The subsets of γδ-TILs from CRC patients were determined by flowcytometry. (**A**) Representative dot plot showed γδ-TILs were dominantly tissue-resident Vδ1 subset rather than circulating Vδ2 subset. (**B**) Scatter diagram summarizes the percentages of Vδ1 positive cells. Data are shown as mean ± SEM; n=3. Unpaired t test was used to compare variables. **P* < 0.05.

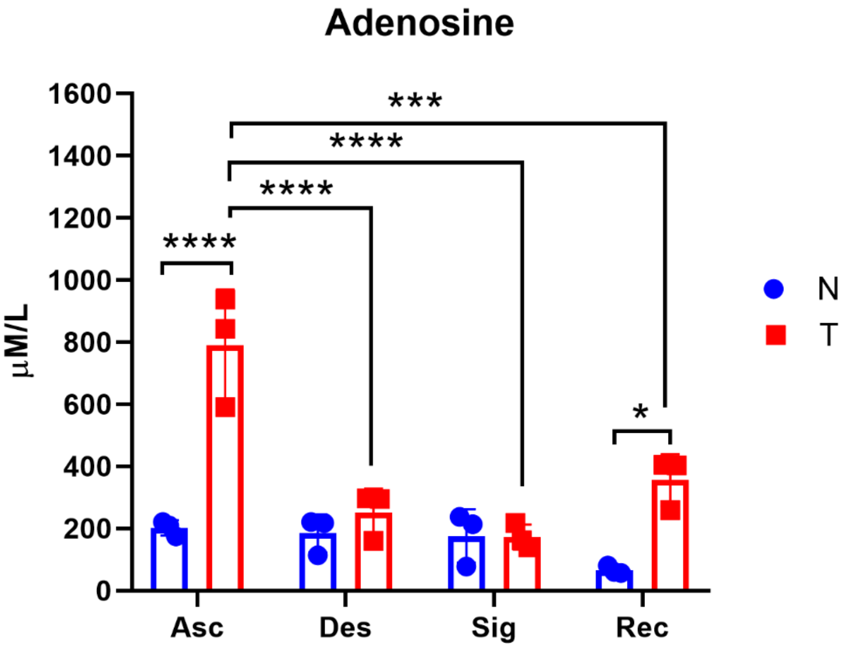

Supplementary Figure 2. Adenosine levels in different colorectal tumors and matched normal tissues. Fresh colorectal cancer and matched normal tissues were homogenized to 1g and cultured in 1640 complete medium for 48 hours. The adenosine level in tissue culture medium was detected by HPLC. Data are shown as mean ± SEM, two-way ANOVA followed by Sidak's multiple comparisons test. n=3. ****P < 0.0001. All data are pooled from at least two independent experiments.

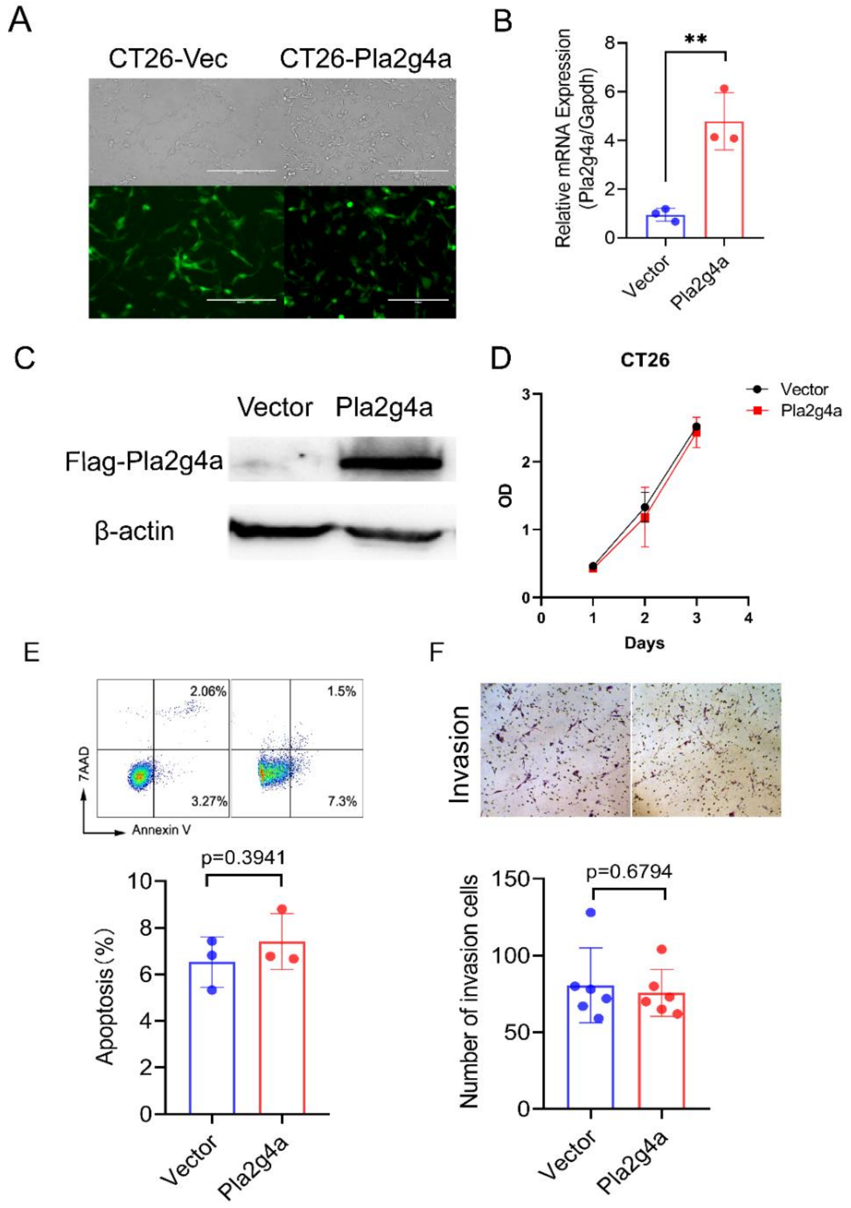

Supplementary Figure 3. Establishment of stable *Pla2g4a*-overexpression cell line. A total of 1 × 10^5 CT26 cells in 1ml 1640 medium with 4ug/ml polybrene were infected with 2x10^6 TU/ml lentivirus. After 48h, 3ug/ml puromycin was added for selection. The *Pla2g4a* overexpression cells (CT26-*Pla2g4a*) and control cells (CT26-Vec) were observed and photographed by fluorescence microscope (**A**). Transfection efficiency was determined by real-time PCR (**B**) and western blot (**C**). Unpaired t test was used to compare variables. **P < 0.01. (**D**) The proliferation of CT26-Vec/*Pla2g4a* was determined by CCK-8. There was no significant difference between the two groups. n=3. (**E**) The apoptosis of CT26-Vec/*Pla2g4a* was determined by flow cytometry. n=3. (**F**) The invasion of CT26-Vec/*Pla2g4a* was shown. Unpaired t test was used to compare variables. n=6. Data are shown as mean ± SEM. All data are pooled from at least two independent experiments.

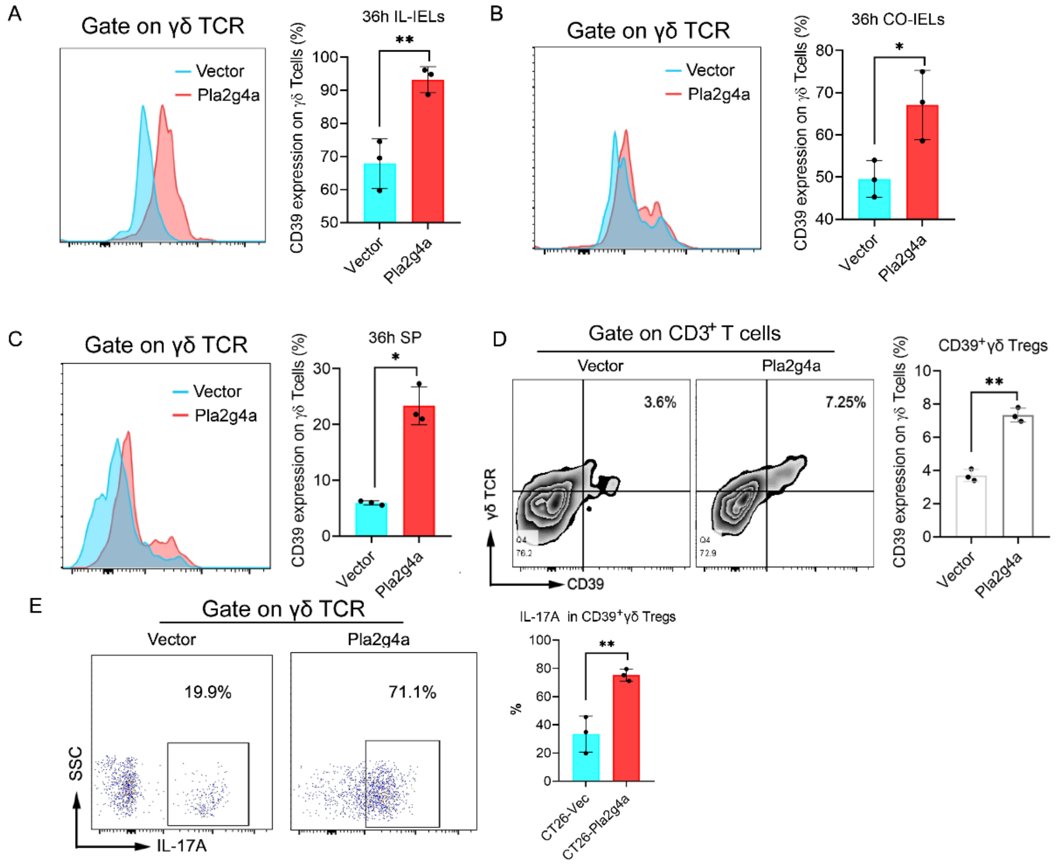

Supplementary Figure 4. Overexpression of *Pla2g4a* in CT26 induced CD39 expression on γδ T cells. CT26-*Pla2g4a* and CT26-Vec cells were cocultured with intraepithelial lymphocytes derived from the ileum (IL-IELs), colon (CO-IELs), or spleen of BALB/c mice. Representative pictures showed the number of CT26 cells in the coculture system after 36 hours. (**A**-**C**) Representative histogram plot and scatter diagram summarize the expression of CD39 on γδ T cells in IL-IELs group (**A**), CO-IELs group (**B**) and splenocytes (**C**) group. (**D**) Representative flow cytometric analysis of CD39^{+}Tregs in CD3^{+} T cells from splenocytes. (**E**) Representative histogram plot and scatter diagram summarize the expression of IL-17A in γδ T cells. Unpaired t test was used to compare variables. n=3. **P* < 0.05; ***P* < 0.01. All of the experiments were repeated three times.

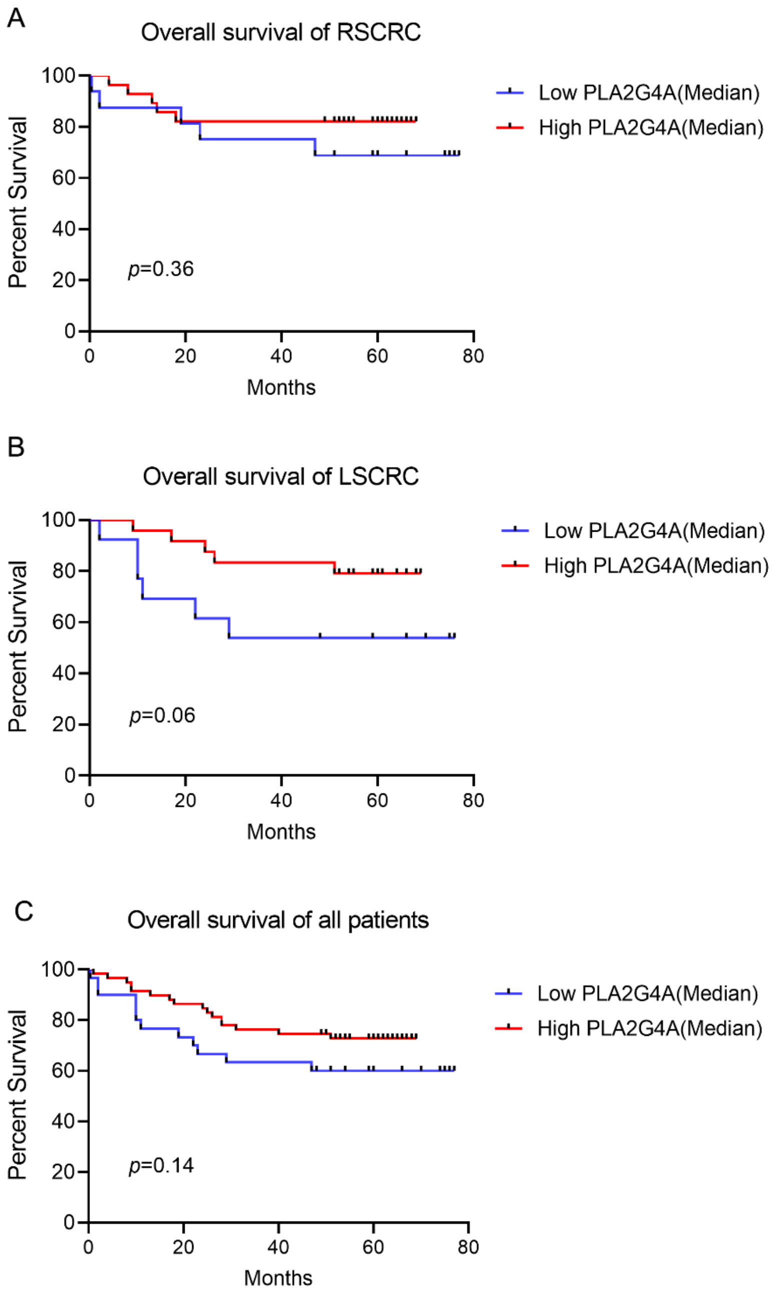

Supplementary Figure 5. The relationship between PLA2G4A expression and the prognosis of patients with colorectal cancer. Kaplan-Meier overall survival curves of stage I-IV RSCRC (**A**), I-IV LSCRC (**B**) and total CRC patients (**C**). Log-rank test were used for analysis. PLA2G4A median level was used as cutoff. RSCRC, n=46; LSCRC, n=48; CRC, n=94.

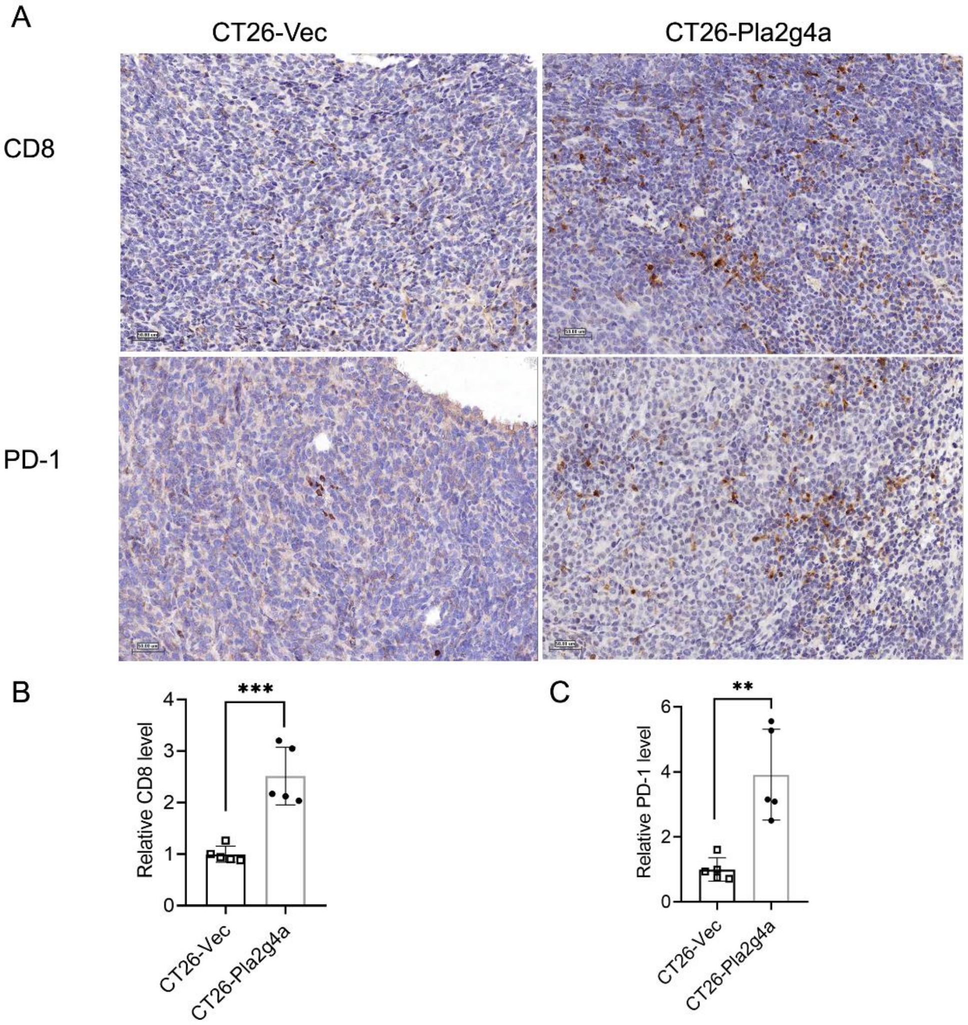

Supplementary Figure 6. The expression of CD8 and PD-1 in the orthotopic murine model of colorectal cancer. (**A**)The representative IHC staining of CD8 and PD-1 expression in orthotopic tumors. Scale bars: 50 μm. (**B**) Statistical analysis of CD8 protein levels in (A). (**C**) Statistical analysis of PD-1 protein levels in (A). Data are shown as mean SEM. Unpaired t test was used to compare variables. n=5. $^{**}P < 0.01$, $^{***}P < 0.001$. All data are pooled from at least two independent experiments.

Suppl. Table 1 Clinicopathological characteristics of patients with different PLA2G4A expression levels undergoing surgery for stage I-IV LSCRC.

		Expression of PLA2G4A (cases)		
Characteristics	Total (cases)	Low	High	*P* value
Total	48	13 (27.1%)	35 (72.9%)	
Age (years)				
＜65	26	7 (26.9%)	19 (73.1%)	0.978
≥65	22	6 (27.3%)	16 (72.7%)	
Gender				
Male	23	7 (30.4%)	16 (69.6%)	0.616
Female	25	6 (24%)	19 (76%)	
Tumor size (cm)				0.758
＜5	20	6 (30%)	14 (70%)	
≥5	27	7 (25.9%)	20 (74.1%)	
Perineuralinvasion				0.672
-	38	10 (26.3%)	28 (73.7%)	
+	9	3 (33.3%)	6 (66.7%)	
Vascularinvasion				0.768
-	34	9 (26.5%)	25 (73.5%)	
+	13	4 (30.8%)	9 (69.2%)	
CD8 expression				0.269
-	27	9 (33.3%)	18 (66.7%)	
+	21	4 (19%)	17 (81%)	
PD-1 expression				0.348
-	36	11 (30.6%)	25 (69.4%)	
+	12	2 (16.7%)	10 (83.3%)	
PD-L1 expression				0.457
-	33	10 (30.3%)	23 (69.7%)	
+	15	3 (20%)	12 (80%)	

Differences between experimental groups were assessed by Pearson ratio χ^2 test. *$P < 0.05$
